# ncRNA-driven regulation of PD-L1 in breast cancer: mechanisms of immune escape and therapeutic opportunities

**DOI:** 10.1007/s10238-026-02252-x

**Published:** 2026-07-19

**Authors:** Amirhossein Mardi, Sajjad Rajabi Moghaddam, Ali Roohi Motlagh, Amirreza Mehmandar-Oskuie

**Affiliations:** 1 Student Research Committee, Tabriz University of Medical Sciences, Tabriz, Iran; 2 Immunology Research Center, Tabriz University of Medical Sciences, Tabriz, Iran; 3 Department of Immunology, Faculty of Medicine, Tabriz University of Medical Sciences, Tabriz, Iran; 4 Student Research Committee, Department of Medical Laboratory Sciences, School of Allied Medical Sciences, Shahid Beheshti University of Medical Sciences, Tehran, Iran; 5 Department of Immunology and Microbiology, Babol University of Medical Sciences, Babol, Iran; 6 Department of Immunology, School of Public Health, Tehran University of Medical Sciences, Tehran, Iran

**Keywords:** PD-L1, Breast cancer, Non-coding RNAs, Immune checkpoint, Tumor microenvironment, Immunotherapy

## Abstract

Programmed death-ligand 1 (PD-L1) is an immune checkpoint molecule important in tumor immune evasion acting primarily by inhibiting T cell activation. In breast cancer, PD-L1 is frequently abnormally expressed and associated with tumor progression, metastasis, and poor prognosis. Recent studies have revealed that non-coding RNAs (ncRNAs) including microRNAs (miRNAs), long non-coding RNAs (lncRNAs), and circular RNAs (circRNAs) are important post-transcriptional regulators of PD-L1. ncRNAs regulate PD-L1 expression through multiple mechanisms, including direct binding to PD-L1 mRNA 3′ UTR, acting as competing endogenous RNAs (ceRNAs), altering transcription factor activity, and altering epigenetic modifications. Dysregulation of ncRNAs not only impact PD-L1-mediated immune escape (i.e., cancer immune evasion), but can also remodel the tumor microenvironment (TME) through impact on immune cell infiltration and activity, and alterations to cytokine production. This review provides an overview of current understanding of ncRNAs regulating PD-L1 in breast cancer, specifically their molecular mechanisms, therapeutic potential, and clinical relevance as biomarkers and/or therapeutic targets. The broad ncRNA-PD-L1 regulatory network is complex, and discoveries in this field may create novel avenues for precision immunotherapy and combination strategies in treating breast cancer.

**Graphical Abstract:**

**Figure:**
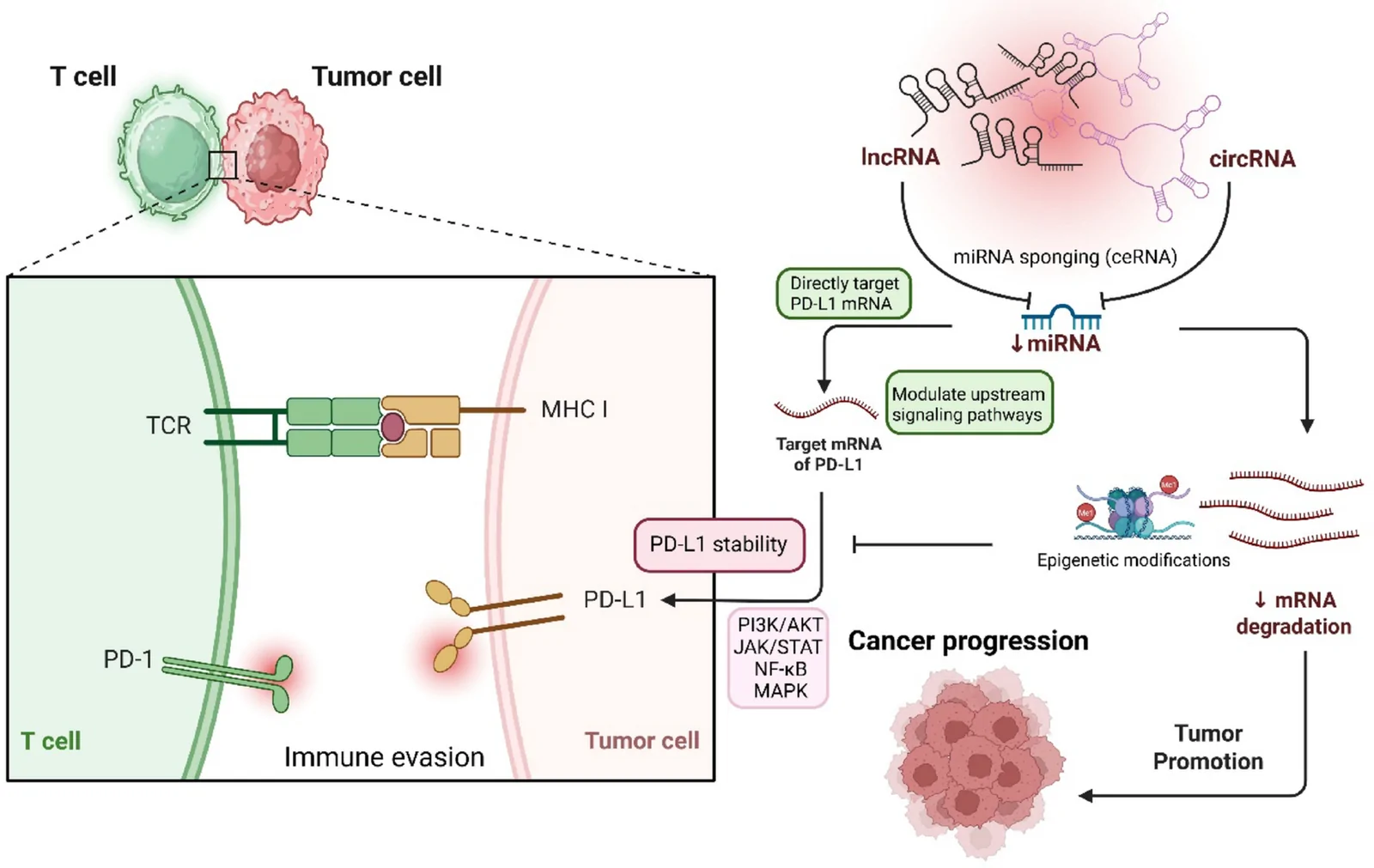

## Introduction

Based on the 2024 American Cancer Society report, breast cancer remains the most commonly diagnosed cancer in women, with a continued rise in incidence and persistent disparities among populations. Although mortality has declined by approximately 44% since 1989 due to advances in early detection and treatment, significant differences in outcomes still exist across racial and ethnic groups [1]. Several factors contribute to the risk of BC, such as sex, increasing age, reproductive history, timing of the first pregnancy, family predisposition, inherited genetic traits, utilization of oral contraceptives, alcohol consumption, and tobacco use [2, 3]. Breast cancer can progress to metastatic disease, in which cancer cells disseminate to distant organs. Metastasis is responsible for the majority of cancer-related deaths, highlighting the importance of early diagnosis for improved prognosis and survival [4]. The management of recurrent BC requires a multidisciplinary strategy that evaluates all possible interventions for optimal results, and current approaches include chemotherapy [5], radiation therapy [6], surgery [7], hormone-based therapies [8] and immunotherapy [9]. Despite their benefits, such therapeutic strategies are frequently hindered by resistance, either innate or acquired, emerging from a range of genetic and epigenetic changes [10, 11]. The immune microenvironment plays a central role not only in cancer but also in various chronic diseases, where dysregulated immune interactions contribute to disease progression and therapeutic resistance [12].

While approximately 1–1.5% of the human genome corresponds to protein-coding regions, large-scale transcriptomic analyses suggest that up to ~ 70–75% of the genome is potentially transcribable, giving rise predominantly to non-coding RNAs [13]. The majority of transcribed products are non-coding RNAs (ncRNAs), which influence mRNA expression and the production of proteins, both through direct and indirect mechanisms. ncRNAs regulate gene activity primarily through functioning like transcription factors, modifying chromatin structure, or participating in post-transcriptional control [14]. Altered gene expression patterns linked to diverse cancers are believed to result, at least in part, from the actions of ncRNAs [15–17]. Consequently, understanding specific ncRNA characteristics can elucidate intricate mechanisms in cancer and enhance its diagnosis and treatment.

Immune checkpoints have a key role in modulating the quality of immune responses. Molecules functioning as co-stimulatory checkpoints (CD27, CD28, CD137, ICOS, 4-1BB, and OX-40) enhance T-cell responses, while co-inhibitory counterparts like PD-1 and CTLA-4 transmit inhibitory cues to restrain T-cell activation and protect against excessive inflammatory or autoimmune reactions [18, 19]. Programmed death ligand 1 (PD-L1), which belongs to the B7 family, suppresses human immune activity via engagement with its receptor, programmed cell death 1 (PD-1) [20]. The PD-1/PD-L1 interaction transmits inhibitory pathways that dampen T-cell activity and play a key role in maintaining immune homeostasis and tolerance [21, 22]. Many studies have shown that the high expression of PD-L1 on cancer cells binds to PD-1 on the surface of T cells, resulting in an immunosuppressive tumor microenvironment and inhibiting T cell activation [23–25]. In recent years, immune checkpoint inhibitors (ICIs), including blocking CTLA-4/CD28 or PD-L1/PD-1 axis have been investigated in various malignancies. ICI combinations have enhanced clinical outcomes across multiple cancer types; nevertheless, they are frequently linked to the emergence of adverse effects in patients [26, 27]. This work investigates the regulatory roles of multiple ncRNA types (miRNAs, lncRNAs, and circRNAs) in the modulation of PD-L1, while also reviewing their possible therapeutic utility in targeting immune escape mechanisms within cancer immunotherapy.

## Non-coding RNAs

ncRNAs constitute the vast majority of the transcriptome and can be categorized into various subtypes according to their length and structural features. Among ncRNAs, ribosome RNA (rRNA) and transfer RNA (tRNA) are the best-characterized types, both of which are indispensable for protein synthesis [28]. Other ncRNAs can be subdivided into two major classes based on their transcript size: small ncRNAs and long ncRNAs. ncRNAs shorter than 200 nucleotides in length are classified as small ncRNAs, which include miRNA, small interfering RNA (siRNA), Piwi-interacting RNA (piRNA), small nucleolar RNA (snoRNA), small nuclear RNA (snRNA) and guide RNA (gRNA). ncRNAs over 200 nucleotides are classified as lncRNAs that significantly influence various pathophysiological processes, particularly in cancer [29, 30]. circRNAs are another subtype of ncRNAs, and their size can range from under 100 nucleotides to over 4 kilobases [31]. Their functions as oncogenes or tumor suppressors have been well established in the regulation of tumor initiation and advancement. Backsplicing, a specific type of alternative splicing, primarily regulates the biogenesis of circRNA [32–34]. The following section provides a brief description of the basic characteristics and mechanisms of miRNA, lncRNA, and circRNA.

### microRNAs

miRNA molecules are small RNA molecules of 21 to 24 nucleotide in length [35]. miRNAs are mainly generated through RNA polymerase II activity in intragenic and intergenic regions; in contrast, polymerase III mediates a limited portion of transcription, typically from Alu-associated miRNA loci [35–37]. miRNAs use seed sequences to bind to miRNA response elements (MREs), predominantly located on the 3′UTRs of mRNAs [38, 39]. miRNAs bind to MRE sites in several different target genes, and mRNAs may have one or several unique MRE sites, making them targets for multiple miRNAs [40, 41]. miRNAs regulate gene expression by binding to the 3′ untranslated regions (3′ UTRs) of mRNAs, leading to translational repression or transcript degradation; however, they can also interact with the 5′-UTRs or even directly associate with promoter regions [42, 43]. These interactions primarily result in target gene expression repression, although some miRNAs contribute to gene expression enhancement. miRNAs belonging to the same family typically co-transcribe and possess a comparable seed sequence, comprising nucleotides 6–8 from the 5′ terminus. Additionally, multiple miRNAs can affect one gene, and one miRNA can inhibit several genes [35, 43].

More than 60% of human genes are thought to be under miRNA regulation, primarily via the recruitment of RNA-induced silencing complexes (RISC) to their target mRNAs [44]. Consequently, even a single miRNA, or a small group of them, can broadly regulate cellular signaling and act in either tumor-suppressive or oncogenic roles [45]. Recent findings highlight the essential functions of specific miRNAs in cancer development, disease progression, and therapeutic response [46, 47]. It was proposed that miRNAs may serve as biomarkers for BC owing to their abnormal expression [48].

### Long non-coding RNAs

lncRNAs are defined as RNA molecules over 200 nucleotides in length without conventional protein-coding capacity, although a subset can generate small peptides or micropeptides [49–51]. They frequently splice similarly to mRNAs, with a 5′ cap and 3′ polyadenylate tail. Some of them operate in the nucleus, whereas others transfer to the cytoplasm [52, 53]. lncRNAs are involved in multiple cellular processes, such as chromatin remodeling, regulation of gene expression, cell proliferation, differentiation, and developmental pathways [54]. Evidence has emerged that lncRNAs are key players in tumor development, where their abnormal expression patterns reflect cancer progression and hold potential as prognostic indicators [55–58].

lncRNAs can be classified based on their genomic location: intergenic, intronic, sense, antisense, and bidirectional [59]. Long intergenic non-coding RNAs, or lincRNAs, originate from intergenic regions that are located between two genes [60]. Intronic lncRNAs are transcribed from intragenic regions (Introns) of protein-coding genes [61]. Sense lncRNAs are transcribed in the 5′ to 3′ direction from the sense strand of protein-coding genes, whereas antisense lncRNAs are transcribed from the antisense strand of protein-coding genes [62, 63]. Bidirectional lncRNAs utilize the same promoter as a protein-coding gene for transcription, occurring in the opposite direction and from both DNA strands [64].

### Circular RNAs

Despite the diversity of circRNA origins, head-to-tail backsplicing primarily produces circRNAs, which are a type of ncRNA that can range from under 100 nucleotides to over 4 kilobases and a covalently closed cyclic structure that does not have a 5′ cap or a 3′ polyadenylate tail [31, 65, 66]. CircRNAs exhibit high stability because of their covalent closed-loop structure, which provides protection against exonucleases [34]. CircRNAs can be classified according to the different splicing sites: Exon circRNA (ecRNA) is produced through exon cyclization from pre-mRNA, is predominantly found in the cytoplasm. Intron circRNA (ciRNA) formed by intron circularization from pre-mRNA, is predominantly located in the nucleus. exon-intron circRNA (elciRNA) is composed of a circRNA that contains both introns and exons due to variable splicing [65, 67, 68].

CircRNAs can modulate different stages of gene expression at the epigenetic, transcriptional, and post-transcriptional levels [69]. CircRNA performs a variety of functions, including inhibiting miRNA activity, which is the most studied mechanism, by acting as miRNA sponges or ceRNAs for protein-coding mRNAs [70, 71]. It also serves as a protein scaffold and binds to RNA-binding proteins (RBPs) to modulate their interaction with target RNA molecules [72, 73]. Additionally, circRNA translates into peptide segments [74, 75], binds to transcription factors, influences host gene transcription [76], and also can influence gene splicing processes [77, 78]. Many studies have reported altered expression patterns of circRNAs in tumor tissues relative to adjacent normal counterparts, where they act either as oncogenes or tumor suppressors in cancer initiation and progression [79–83]. Despite our knowledge of the dysregulated expression of circRNAs and other ncRNAs in various cancers, more research is necessary to fully comprehend their role in pathogenesis and therapeutic applications.

## PD-L1 in immune evasion

T-cell-mediated immunity is important for keeping homeostasis because it targets and kills pathogens and abnormal cells, such as cancer cells. However, when T cells become too active and out of control, they may also attack normal cells [84]. There are two types of signals that T cells need to fully activate: antigen receptor signaling and CD28 costimulatory signaling. Immune checkpoint proteins control how T cells work when the body is working normally; Therefore, immune checkpoint proteins suppress autoimmunity and prevent autoimmune diseases. However, evidence indicates that cancer cells exploit immune checkpoint proteins on their surface as a strategy to escape immune surveillance [85–87].

Programmed death-ligand 1 (PD-L1), a member of the B7 family of T-cell co-inhibitory molecules, functions as an immune checkpoint by binding to its receptor, programmed cell death 1 (PD-1) [88]. PD-1 has two known ligands, PD-L1 and PD-L2. Upon PD-1/PD-L1 engagement, tyrosine residues within the immunoreceptor tyrosine-based switch motif (ITSM) of the PD-1 cytoplasmic tail become phosphorylated, facilitating the recruitment of Src homology 2 domain-containing phosphatase-2 (SHP-2). SHP-2 subsequently dephosphorylates downstream signaling molecules such as spleen tyrosine kinase (Syk) and phosphoinositide-3 kinase (PI3K), thereby suppressing T-cell biological functions, including proliferation, cytokine production, and cytotoxic T lymphocyte (CTL) activity [23, 89, 90].

PD-L1 serves as the primary ligand, and its elevated expression in cancer cells enables tumors to suppress the proliferation and survival of tumor-specific T cells [91–94]. As a result, T-cell cytotoxic activity in the TME is diminished, promoting their exhaustion and apoptosis, thereby allowing tumor cells to evade immune surveillance [95, 96]. Employing antibodies that block immune checkpoint pathways in T cells represents an immunotherapeutic strategy with substantial clinical benefits [97]. The U.S. Food and Drug Administration (FDA) approved several commercially available anti-PD-L1 monoclonal antibodies, including atezolizumab, avelumab, and duravulumab [98–101]. The goal of tumor immunotherapy is to inhibit immune checkpoint proteins and enhance the ability of T cells against tumor [102]. Identifying biomarkers is necessary to personalize treatments and achieve the best possible treatment effect, as some patients have low PD-L1 expression [103, 104]. Although PD-1/PD-L1 blockade is an appealing therapeutic approach in cancer immunotherapy, reports indicate that 30% to 60% of cancer patients develop primary and adaptive resistance to anti-PD-1/PD-L1 therapy and clinical responses significantly differ among various tumor entities due to tumor heterogeneity and numerous other factors [105–107].

## Progression of breast cancer mediated by miRNAs/PD-L1

BC exhibits significant genomic and clinical heterogeneity. Epigenetic alterations, such as abnormal production of miRNAs, which deactivate several genes, are present in the molecular features of breast cancer cells [108–110]. miRNAs are important in the development of breast cancer, and PD-L1-mediated immune suppression is linked to deregulation of miRNAs [111]. Our previous study demonstrated that specific circulating miRNAs, including miR-4443, miR-572, and miR-150-5p, serve as potential diagnostic biomarkers in breast cancer [112]. These findings further support the broader role of miRNAs as clinically relevant regulators in breast cancer, which may also extend to immune modulation and tumor microenvironment interactions. Tumor-derived miRNAs play a key role in remodeling the tumor microenvironment by regulating innate immune cells such as macrophages, dendritic cells, myeloid-derived suppressor cells, and natural killer cells [113]. In breast cancer, the PD-L1/PD-1 functions as an inhibitory immunological checkpoint axis [114]. Through a complicated method, the miRNA network regulates genes associated with immunological checkpoints and affects the levels of PD-L1 and PD-1 [115–117]. Clinical and molecular subtypes [118], progression [119], prognosis [120], and oncogene expression [121] all have been associated with miRNA expression in breast tumor.

**Fig. 1:**
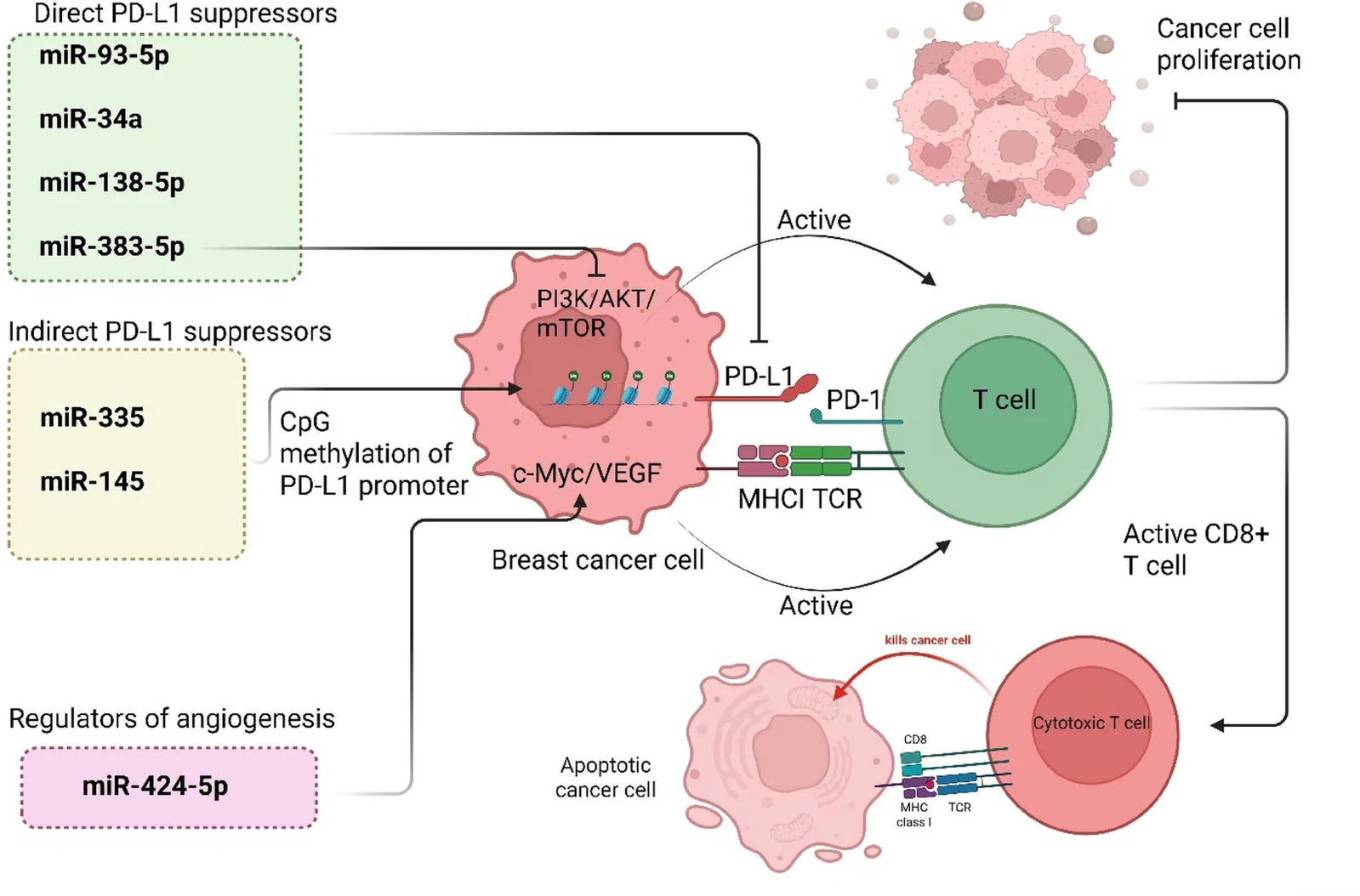
miRNA-mediated regulation of PD-L1 signaling in breast cancer progression. In breast cancer cells, altered expression of miRNAs (e.g., miR-93-5p, miR-34a, miR-335, miR-145, miR-424-5p, miR-138-5p, and miR-383-5p) influences the PD-L1/PD-1 immune checkpoint axis through direct targeting or indirect modulation of associated oncogenic pathways (e.g., CCND1, c-Myc, VEGF, PI3K/AKT/mTOR). These miRNAs can promote apoptosis, cell cycle arrest, and reduction in proliferation. Additional regulatory layers include epigenetic alterations such as CpG island hypermethylation. The net outcome is modulation of the tumor microenvironment, including suppression or activation of cytotoxic T cell responses, which ultimately affects breast cancer progression, prognosis, and metastatic spread

According to the reported findings, miR‑93‑5p directly binds to and post‑transcriptionally regulates the PD‑L1/CCND1 axis. Reduced miR‑93‑5p expression in TNBC cells (MDA‑MB‑231) is associated with elevated levels of both PD‑L1 and CCND1, whereas restoration of miR‑93‑5p leads to suppression of their expression. These results indicate that miR‑93‑5p concurrently targets PD‑L1 and CCND1, thereby modulating pathways involved in tumor cell proliferation and immune regulation in breast cancer [122]. Based on the reported results, p53 negatively regulates PD‑L1 expression through transcriptional activation of miR‑34a in triple‑negative breast cancer cells. The study demonstrated that miR‑34a directly targets PD‑L1, leading to a reduction in its expression at both mRNA and protein levels. Restoration of p53 increased miR‑34a expression, which in turn suppressed PD‑L1. Functionally, miR‑34a overexpression inhibited cell proliferation and migration, induced apoptosis, and reduced cell viability. These findings indicate that the p53/miR‑34a axis restrains tumor growth by downregulating PD‑L1 in TNBC cells [123]. Tavazoie et al. [109] identified miR‑335 as an endogenous suppressor of breast cancer metastasis, showing that re‑expression of miR‑335 in highly metastatic breast cancer cells markedly reduced metastatic colonization of the lung and bone. In addition, analysis of the miR‑143/145 cluster demonstrated that miR‑145 exerts inhibitory effects on breast cancer cell proliferation and invasion in vitro and is downregulated in malignant compared with benign breast tissue [124]. Furthermore, aberrant promoter hypermethylation of miR‑335 and miR‑145 has been reported to correlate with reduced miRNA expression and increased PD‑L1 levels in breast tumors. Restoration of these miRNAs is associated with decreased proliferation and induction of cell cycle arrest and apoptosis in breast cancer cells [125]. According to Dastmalchi et al., [126] miR‑424‑5p exerts tumor‑suppressive effects in MDA‑MB‑231 cells by directly targeting PD‑L1 and modulating the PTEN/PI3K/AKT/mTOR signaling pathway. Its overexpression reduced cell viability, induced G2/M cell‑cycle arrest, and promoted apoptotic and autophagic responses. In addition, transfection of miR‑424‑5p decreased c‑Myc protein levels, a transcription factor involved in cell growth regulation, and is associated with reduced VEGF expression, a key mediator of tumor angiogenesis [127, 128]. Collectively, these findings indicate that miR‑424‑5p suppresses breast cancer cell progression through coordinated regulation of PD‑L1 and oncogenic signaling pathways. miR‑138‑5p has been shown to exert pro‑apoptotic effects in breast cancer cells by increasing the expression of caspase‑9 and caspase‑3 and inducing cell‑cycle arrest at the sub‑G1 phase. Ectopic expression of miR‑138‑5p in MDA‑MB‑231 cells significantly reduced PD‑L1 levels. Through downregulation of PD‑L1, miR‑138‑5p interferes with the PD‑1/PD‑L1 axis, thereby attenuating T‑cell exhaustion and modulating T‑cell cytokine production. These findings indicate that miR‑138‑5p contributes to both apoptotic regulation and immune response modulation in breast cancer [129]. miR‑383‑5p has been reported to enhance antitumor immune activity by promoting the secretion of pro‑inflammatory cytokines from T cells following transfection. In breast cancer tissues, PD‑L1 expression is elevated, whereas miR‑383‑5p levels are reduced compared with non‑malignant counterparts. Mechanistically, miR‑383‑5p suppresses tumor progression by inhibiting the PI3K/AKT/mTOR signaling pathway and downregulating PD‑L1 expression. Through modulation of this pathway and the PD‑1/PD‑L1 axis, miR‑383‑5p contributes to the establishment of a pro‑inflammatory tumor microenvironment that may support immune‑mediated tumor elimination [111] **(**Table 1**) (**Fig. 1**)**. Taken together, the evidence indicates that dysregulated miRNAs shape breast cancer progression by fine‑tuning PD‑L1 expression and its upstream signaling networks. By coordinating immune checkpoint control with pathways governing proliferation, survival, and metastasis, the miRNA–PD‑L1 interplay represents a central mechanism connecting tumor biology with immune modulation. Moreover, although these findings highlight the regulatory role of miRNAs in PD-L1 modulation, most studies are based on in vitro models, and their clinical relevance remains to be fully validated.

**Table 1:** PD-L1-regulating miRNAs in breast cancer progression

miRNA	Target(s) / Pathway(s)	Effect on PD-L1	Additional Effects on Cancer Cells	Other Pathways/Effects	Experimental Context / Model	Refs.
miR-93-5p	PD-L1, CCND1	↓ PD-L1 via PD-L1/CCND1 axis	Controls tumor growth; regulates immune response; affects distribution in cytoplasm, nucleus, and membrane	Alters CCND1 localization (cytoplasm, nucleus)	MDA-MB-231 (TNBC) vs. HBL-100 (normal breast cells)	[122]
miR-34a	PD-L1	↓ PD-L1	Suppresses migration; induces cytotoxicity & apoptosis in TNBC	p53-mediated induction	TNBC models	[123]
miR-335	Metastasis-related genes	↓ PD-L1 (via demethylation & expression restoration)	Suppresses lung & bone metastasis; cell cycle arrest; apoptosis	Suppresses metastasis genes	Breast cancer tissues & cell lines	[109, 125]
miR-145	Invasion/migration genes	↓ PD-L1 (via demethylation & expression restoration)	Inhibits invasion/migration; cell cycle arrest; apoptosis	Controls invasion, migration genes	Breast cancer tissues & cell lines	[124]
miR-424-5p	c-Myc, VEGF; apoptotic/autophagic pathways	Not directly stated	↓ Cell viability; ↑ G2/M arrest; ↓ c-Myc & VEGF	Regulates apoptosis & autophagy	MDA-MB-231	[126–128]
miR-138-5p	PD-L1	↓ PD-L1	↑ Caspase-3 & -9; apoptosis; prevents T cell exhaustion; disrupts PD-L1/PD-1	Activates caspase-3, -9	MDA-MB-231	[129]
miR-383-5p	PD-L1, PI3K/AKT/mTOR	↓ PD-L1	↑ Pro-inflammatory cytokine secretion; tumor suppression via PI3K/AKT/mTOR blockade	Induces pro-inflammatory cytokines	Breast cancer tissues & T cells	[111]

## Progression of breast cancer mediated by lncRNAs/PD-L1

Accumulating evidence supports a pivotal role for lncRNAs in breast cancer progression, particularly through modulation of immune checkpoint pathways. Several lncRNAs enhance PD‑L1 expression by functioning as ceRNAs or by influencing transcriptional and epigenetic regulators, thereby facilitating tumor immune escape and metastatic potential [130, 131]. Consequently, the lncRNA/PD‑L1 regulatory network has emerged as a promising target for immunotherapeutic intervention. Notably, in TNBC, lncRNAs have been implicated in tumor initiation, invasion, metastasis, recurrence, and resistance to chemotherapy, underscoring their clinical relevance [132]. Some lncRNAs may be important to cancer immunology, including infiltration of immune cells, immunological evasion, and antigen presentation, according to new research [133, 134]. Furthermore, lncRNAs in the cytoplasm have a major role for regulating protein translation and post-transcriptional gene levels, including mRNA or protein stability via the ceRNA mechanism [135, 136].

**Fig. 2:**
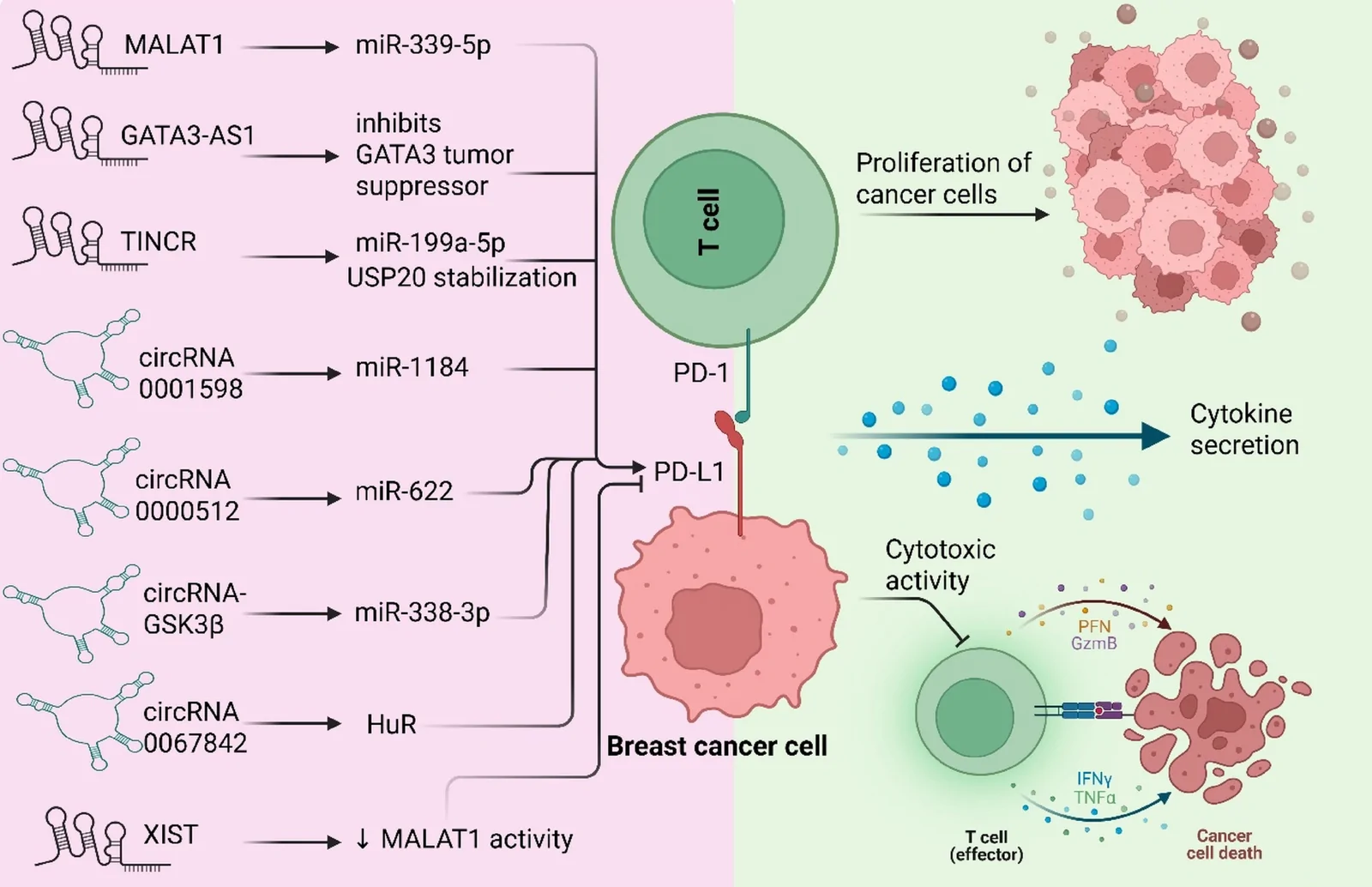
Mechanistic roles of lncRNAs and circRNAs in regulating PD-L1 expression and immune escape in breast cancer. The schematic illustrates how oncogenic lncRNAs (MALAT1, etc.) and circRNAs (circRNA_0001598, circRNA_0000512, etc.) upregulate PD-L1 expression in breast cancer cells through various mechanisms, including miRNA sponging, inhibition of tumor suppressors, stabilization of mRNA, and modulation of ubiquitination pathways. PD-L1 on tumor cells binds PD-1 on T cells, leading to impaired cytotoxic activity and cytokine secretion, thereby promoting tumor cell proliferation and immune evasion. Conversely, the tumor-suppressive lncRNA XIST inhibits MALAT1 activity, reducing PD-L1 expression

In a variety of cancer forms, the lncRNA MALAT1 has a major carcinogenic role and investigations shown that breast cancer tissues have markedly elevated MALAT1 mRNA expression [137–139]. In this regard, according to Samir et al., MALAT1 is markedly overexpressed in breast cancer tissues, particularly in TNBC, where its elevated levels are associated with increased cell proliferation and invasion as well as unfavorable clinicopathological characteristics and reduced overall survival. Importantly, the study demonstrated a clear regulatory link between MALAT1 and PD‑L1, as MALAT1 knockdown significantly decreased PD‑L1 expression. These findings indicate that MALAT1 actively sustains PD‑L1–mediated signaling, highlighting its contributory role in immune regulatory mechanisms and breast cancer progression [137]. XIST and TSIX, two lncRNAs involved in X‑chromosome inactivation, have also been implicated in breast cancer and PD‑L1 regulation. An inverse relationship between XIST and PD‑L1 expression has been reported, as silencing XIST leads to a significant increase in PD‑L1 levels. In contrast, downregulation of TSIX resulted in a marked decrease in PD‑L1 expression [140]. Moreover, XIST has been shown to modulate the PD‑L1 signaling pathway partly through counteracting the oncogenic activity of MALAT1 [137]. Consistent with their regulatory interplay, TSIX negatively regulates XIST, whereas XIST exhibits tumor‑suppressive properties in breast cancer [141–143]. GATA3‑AS1 is a recently characterized lncRNA that is significantly upregulated in triple‑negative breast cancer (TNBC) tissues and cell lines. Functional Studies have indicated that silencing GATA3‑AS1 suppresses TNBC cell proliferation and migration while promoting apoptosis, suggesting an oncogenic role in this subtype. Mechanistically, GATA3‑AS1 enhances immune evasion by facilitating CSN5‑mediated deubiquitination and stabilization of PD‑L1, thereby sustaining PD‑L1 expressions [144]. In addition, GATA3‑AS1 regulates its neighboring gene, GATA3, at the protein level by promoting its ubiquitination and destabilization. Given the tumor‑suppressive function of GATA3 in human malignancies, its degradation further supports the pro‑tumorigenic activity of GATA3‑AS1 in TNBC progression [145, 146]. TINCR is positively correlated with PD‑L1 expression and contributes to breast cancer cell proliferation and metastasis in both in vitro and in vivo models. Mechanistically, TINCR functions through dual regulatory mechanisms. In the cytoplasm, it acts as a competing endogenous RNA by sponging miR‑199a‑5p, thereby enhancing the stability of USP20 mRNA. In the nucleus, TINCR interacts with DNMT1 to promote methylation of the miR‑199a‑5p promoter, suppressing its transcription. The resulting upregulation of USP20 prevents PD‑L1 ubiquitination, leading to PD‑L1 stabilization and accumulation, which supports tumor progression and immune evasion [147]. In HER2‑positive breast cancer, ZNF649‑AS1 is elevated in trastuzumab‑resistant cases and promotes resistance to therapy. Mechanistically, ZNF649‑AS1 binds to PRPF8 and regulates alternative splicing of EXOC7, increasing the EXOC7‑S isoform. Functional studies showed that silencing ZNF649‑AS1 reduced trastuzumab resistance and decreased tumor‑associated macrophage (TAM)–dependent PD‑L1 expression. Likewise, EXOC7‑S knockdown suppressed PD‑L1 upregulation, whereas its overexpression reversed the effects of ZNF649‑AS1 depletion, linking this lncRNA to immune‑mediated therapeutic resistance [148]. **(**Table 2**) (**Fig. 2**)**. Collectively, these findings highlight that multiple lncRNAs contribute to breast cancer progression and therapeutic resistance by modulating PD‑L1 expression through diverse mechanisms, including alternative splicing, ceRNA activity, epigenetic regulation, and protein stabilization.

**Table 2:** Roles of lncRNAs in Breast Cancer and Their Regulation of PD-L1/Immune Escape

lncRNA	Expression Pattern	Functional Role	Mechanism(s) of Action	Effect on PD-L1 / Immune Escape	Refs
MALAT1	Upregulated	Promotes proliferation, invasion	Correlates with tumor stage, histological grade, lymph node status; oncogenic	Knockdown ↓ PD-L1 expression; reduces immune evasion	[137, 138, 192, 193]
XIST	Downregulated (tumor suppressor)	Tumor suppressor; inhibits MALAT1	Regulates PD-L1 signaling via MALAT1 inhibition	Knockdown ↑ PD-L1 expression	[140]
TSIX	Not specified	Negative regulator of XIST	Knockdown of TSIX ↑ XIST	Knockdown ↓ PD-L1 expression	[140, 143]
GATA3-AS1	Upregulated	Promotes proliferation, migration; inhibits apoptosis	Modulates CSN5-mediated PD-L1 deubiquitination; regulates nearby GATA3 gene by promoting its ubiquitination	Knockdown ↓ PD-L1 expression; enhances immune response	[144]
WEE2-AS1	Upregulated	Promotes proliferation, migration, invasion; inhibits apoptosis	Not specified in detail	Not specified	[194]
KLHDC7B	Upregulated	Promotes migration, resistance to cell death	Not specified	Not specified	[195]
MIR100HG	Upregulated	Oncogenic	Forms triplex with p27 locus	Not specified	[196]
TINCR	Upregulated	Promotes proliferation, metastasis	Upregulates PD-L1 via ceRNA sponging of miR-199a-5p and transcriptional repression of miR-199a-5p (via DNMT1-mediated methylation); stabilizes USP20 mRNA	↑ PD-L1 expression; knockdown enhances PD-L1 inhibitor efficacy	[147]

## Progression of breast cancer mediated by circRNAs/PD-L1

CircRNAs in breast tumors may act as oncogenes or tumor suppressors. They affect multiple biological processes, including apoptosis and cell proliferation [33, 149]. For example, circBCBM1 (hsa_circ_0001944) is upregulated in breast cancer brain metastasis and exhibits oncogenic activity. It promotes proliferation, migration, tumor growth, and brain metastatic colonization by acting as a miR‑125a sponge, thereby upregulating BRD4 and activating downstream SHH/MMP9 signaling. Elevated circBCBM1 correlates with shorter brain metastasis‑free survival [150]. On the other hand, recent evidence indicates that specific circRNAs contribute to tumor progression and immune evasion by regulating PD‑L1 expression, thereby influencing the PD‑1/PD‑L1 immune checkpoint axis in breast cancer. Dong et al. indicated that hsa_circ_0067842 contributes to breast cancer progression by regulating PD‑L1 stability through intermediary molecules [151]. This circRNA interacts with the RNA‑binding protein HuR and promotes its nuclear translocation, thereby enhancing the stability of CMTM6 mRNA [152]. Given that CMTM6 acts as a key post‑translational regulator of PD‑L1 by preventing its ubiquitination and degradation, increased CMTM6 expression results in stabilization of PD‑L1 protein. Accordingly, modulation of hsa_circ_0067842 expression leads to corresponding alterations in PD‑L1 protein levels, linking this circRNA to PD‑L1–mediated immune escape and enhanced invasive behavior in breast cancer cells [151]. Likewise, circ‑0000512 regulates PD‑L1 protein stability through a CMTM6‑dependent mechanism in TNBC. While miR‑622 directly suppresses CMTM6 expression at both mRNA and protein levels, circ‑0000512 downregulates miR‑622, thereby relieving its inhibitory effect on CMTM6. Given that miR‑622 is markedly reduced in TNBC, circ‑0000512 further enhances CMTM6 expression, which in turn limits PD‑L1 ubiquitination and stabilizes PD‑L1 protein. Notably, circ‑0000512 overexpression did not alter PD‑L1 mRNA levels but increased PD‑L1 protein abundance, whereas its silencing promoted PD‑L1 ubiquitination, reduced proliferation and migration, and induced TNBC cell death [153]. circRNA‑GSK3β is highly expressed in breast cancer cells and has been proposed as a potential biomarker for diagnosis and prognosis. Functionally, it acts as a ceRNA by binding miR‑338‑3p, thereby relieving repression of its target gene, PRMT5. Upregulation of PRMT5 leads to increased PD‑L1 expression through enhanced recruitment of H3K4me3 at the PD‑L1 promoter region. Through this mechanism, circRNA‑GSK3β promotes immune escape while also augmenting the proliferative, migratory, and invasive capacities of breast cancer cells [154]. In addition, circRNA-ATAD2 facilitates tumor immune evasion through suppression of CD8⁺ T cell cytotoxic function and increased PD-L1 mRNA stability and expression. Through stabilization of PD-L1 transcripts, elevated circRNA-ATAD2 expression compromises CD8⁺ T cell-mediated immune defense mechanisms in breast cancer [155]. circRNA_0001598 functions as a molecular regulator of miR‑1184 in breast cancer. miR‑1184 directly targets PD‑L1, thereby suppressing its expression. By sequestering miR‑1184, circRNA_0001598 relieves this inhibition and enhances PD‑L1 levels. Restoration of miR‑1184 abrogated the pro‑tumorigenic effects of circRNA_0001598, including increased breast cancer cell proliferation, elevated PD‑L1 expression, and resistance to CD8⁺ T‑cell–mediated cytotoxicity. These findings highlight the functional significance of the circRNA_0001598/miR‑1184/PD‑L1 axis in breast cancer progression and immune evasion [156]. In addition, CircRNA-WWC3 enhances both the transcriptional activity and extracellular release of IL-4 within breast cancer cells. Beyond promoting the migratory behavior of breast cancer cells, elevated secretion of IL-4 by these cells contributes to the upregulation of PD-L1 in M2-polarized macrophages and fosters their immunosuppressive polarization within the TME. Moreover, aberrant upregulation of IL-4 in breast carcinoma cells has been shown to enhance the expression of PD-L1, thereby facilitating tumor immune evasion mechanisms and contributing to the immunosuppressive landscape of breast cancer [157] **(**Table 3**) (**Fig. 2**)**. Overall, circRNAs play a significant role in breast cancer progression by regulating PD‑L1 expression through miRNA sequestration and modulation of PD‑L1 stability. This regulatory network contributes to immune evasion and tumor aggressiveness, highlighting the importance of the circRNA/PD‑L1 axis in disease development.

**Table 3:** Circular RNAs regulating CMTM6/PD-L1 and Immune Escape in Breast Cancer

circRNA	Expression Pattern	miRNAs	Functional Role	Mechanism(s) of Action	Effect on PD-L1 / Immune Escape	Refs
hsa_circ_0067842	Upregulated	-	Promotes invasion & migration	Binds HuR → ↑ CMTM6 mRNA stability → inhibits PD-L1 ubiquitination → prevents PD-L1 degradation	↑ PD-L1 protein expression	[151]
circRNA-0000512	Upregulated	miR-622	Promotes proliferation, migration; inhibits apoptosis	Sponges miR-622 → ↑ CMTM6 expression → inhibits PD-L1 ubiquitination	↑ PD-L1 protein (no change in mRNA)	[153]
circRNA-GSK3β	Upregulated	miR-338-3p	Promotes proliferation, migration, invasion	Sponges miR-338-3p → ↑ PRMT5 → ↑ H3K4me3 enrichment at PD-L1 promoter	↑ PD-L1 transcription	[154]
circRNA-ATAD2	Upregulated	-	Inhibits CD8⁺ T-cell cytotoxicity	Stabilizes PD-L1 mRNA	↑ PD-L1 protein → immune escape	[155]
circRNA_0001598	Upregulated	miR-1184	Promotes proliferation; inhibits CD8⁺ T-cell cytotoxicity	Sponges miR-1184 → releases PD-L1 from repression	↑ PD-L1 expression	[156]
circRNA-WWC3	Upregulated	-	Promotes migration; modulates TME	↑ IL-4 secretion → ↑ PD-L1 in M2 macrophages + ↑ M2 polarization; ↑ PD-L1 in BC cells	↑ PD-L1 via IL-4–mediated pathway	[157]

Although individual ncRNAs have been extensively characterized in regulating PD-L1 expression, accumulating evidence suggests that these molecules rarely function in isolation. Instead, they form complex and dynamic regulatory networks, particularly through ceRNA mechanisms, which integrate multiple layers of gene regulation [158]. In ceRNA networks, lncRNAs and circRNAs act as molecular sponges for miRNAs, thereby relieving miRNA-mediated repression of PD-L1 [159]. Importantly, these ncRNA-mediated networks are tightly integrated with upstream transcriptional regulators [160]. Although individual ncRNAs have been extensively characterized in regulating PD-L1 expression, accumulating evidence suggests that these molecules rarely function in isolation. Instead, they form complex and dynamic regulatory networks, particularly through ceRNA mechanisms.

## Therapeutic potential of ncRNAs

### miRNAs

miRNAs play a critical role in regulating tumor behavior within the TME, influencing not only cancer cells but also stromal components such as immune cells. Through these interactions, miRNAs contribute to tumor progression, immune modulation, and metastatic dissemination. A theoretical basis for creating novel treatment strategies is provided by the regulatory role of miRNAs in the TME. Current therapeutic strategies involving miRNAs in oncology primarily encompass three key approaches: reactivation of tumor-suppressive miRNAs, inhibition of oncogenic miRNAs, and exploitation of miRNAs as nanocarriers for targeted drug delivery [161]. For instance, enhancing the expression of tumor-suppressive microRNAs, such as miR-34a, may impede osteosarcoma progression by downregulating multiple oncogenic targets, including Notch1 and Bcl-2 [162]. Similarly, another important therapeutic approach is to inhibit the function of oncogenic miRNAs. Antisense oligonucleotides (ASOs) or miRNA inhibitors may be used to target miR-21, which is overexpressed in a variety of malignancies, in order to lower its levels and inhibit tumor progression [163]. Two major challenges in the clinical implementation of miRNA-based therapies are limited bioavailability and off-target effects [164]. Bioavailability, is due to any synthetic miRNA mimics must be taken up and processed inside the cell properly, based on molecularly designed structure (164). Conversely off-target effect is due to the mechanism of intrinsic miRNA activity, the inherently imperfect binding affinity creates functional binding to multiple mRNA transcripts, that would not otherwise be changed [165]. This precarious binding may also induce alterations to regulation of genes unrelated to the intended therapeutic target being manipulated which might also produce unintended biological results. miR-155 stimulation of enhancing the immuno-stimulatory aspects of tumor-associated DCs may be potentially compromised if it is poorly designed or improperly delivered, as it could interfere with other critical signaling cascades producing side effects [165, 166]. The delivery process itself is another significant barrier. Native miRNAs, when administered without chemical modification, are rapidly degraded by endogenous nucleases, resulting in a shortened functional half-life and an elevated risk of unintended off-target gene silencing. Therefore, it is critical to develop delivery platforms that protect miRNAs from degradation, and allow for selective delivery to tissue of interest [167, 168] **(**Table 4**).**

**Table 4:** Challenges and translational limitations of ncRNAs

ncRNA type	Key challenges	Translational limitations
miRNA	Off-target effects, instability	Delivery, specificity
lncRNA	Low conservation, heterogeneity	Targeting, validation
circRNA	Poor synthesis, cost	Scalability, clinical translation

### lncRNAs

Within the TME, lncRNAs modulate tumor progression and therapeutic efficacy by influencing immune cell dynamics, including their activation status, infiltration capacity, and the expression of immune checkpoint molecules [169]. LncRNAs serve multifaceted roles within TME therapeutics, functioning as prognostic biomarkers, modulators of immunotherapeutic response, and potential targets for therapeutic intervention [170, 171]. They provide vital information on patient survival and responses to certain therapies. Elevated lncRNA HOTAIR expression is associated with poorer outcomes and a quicker rate of disease progression [172]. LncRNAs are needed for tumor development, metastasis, and tumor progression, but numerous challenges impede their translation into clinical applications. Initially, the tissue-specific expression patterns and heterogeneous transcriptional profiles of lncRNAs pose significant challenges to their application as reliable diagnostic biomarkers or therapeutic targets [173]. A second challenge is achieving lncRNA delivery into target cells without compromising lncRNA functional capabilities [174] **(**Table 4**)**. In addition to these factors, the lncRNAs as therapeutics are also dependent on achieving high stability and target specific selectivity, yet considerable improvement is still needed on biotechnologies focused on selective lncRNA recognition and degradation [175, 176]. Advances in carriers (e.g., nanoparticle systems, liposomal delivery systems, viral vectors) have improved opportunities for highly selective delivery [177, 178]. Also, strategies using siRNA and ASOs against lncRNAs have demonstrated promising results in preclinical studies, including a favorable safety profile [179].

### circRNAs

CircRNAs’ functions in the initiation, spread, and treatment of cancer have attracted a lot of study. These molecules are essential for many biological functions, including controlling the expression of genes and the activity of proteins [180]. Recent evidence shows that circPLK1 is significantly upregulated in triple-negative breast cancer and contributes to anthracycline resistance. Mechanistically, circPLK1 promotes tumor cell survival by sponging miR-940 and upregulating ETS1, leading to enhanced proliferation, drug resistance, and poor prognosis in TNBC patients [181]. CircRNAs are excellent candidates for biomarkers and therapeutic targets because of their great stability, conservation, and tissue specificity [182]. By boosting CD4^+^ and CD8^+^ T cells, improving antigenic cross-presentation, and activating dendritic cells, circRNAs may strengthen the anti-tumor immune response [183]. These findings imply that circRNAs may be useful in cancer immunotherapies. In addition to T cell-mediated immune checkpoints, natural killer cells also express inhibitory receptors such as NKG2A, KIRs, and TIGIT, which play critical roles in tumor immune evasion and have recently emerged as promising therapeutic targets in cancer immunotherapy [184]. Moreover, circRNA-based vaccine platforms have demonstrated robust antigen-specific T cell activation and pronounced antitumor efficacy [185]. The clinical translation of circRNAs has its own unique limitations. The limited evolutionary conservation of circRNAs, estimated at only 10%–20% between humans and murine models—poses challenges to the translational applicability of animal-derived data in human disease contexts [186]. Additionally, technical barriers to circRNA clinical usage, such as poor synthesis routes to obtain circRNAs, high costs for enzymatic reagents, etc., limit development of circRNA-based biomarkers and cancer immunotherapies on a large scale [187]. An emerging therapeutic strategy involves integrating circRNA-based interventions with immune checkpoint blockade approaches, specifically PD-1/PD-L1 inhibitors, to potentiate antitumor immune responses and counteract immune evasion mechanisms. For example, circRHBDD1 has been shown to promote immune escape in gastric cancer through the IGF2BP2/PD-L1 axis and can serve as a potential nanomedicine target [188, 189]. Moreover, oncogenic circRNAs such as circKIF4A and circBACH1 promote metastatic progression through activation of PI3K/AKT and STAT3 signaling pathways, whereas tumor-suppressive circRNAs such as circFOXO3 and circNFIB inhibit metastasis by regulating angiogenesis and metabolic reprogramming [190].

## Conclusion and future directions

Despite their therapeutic potential, the translation of ncRNAs (miRNAs, lncRNAs, circRNAs) into clinical applications remains limited by biological and technical challenges. For miRNAs, it is important that they possess the optimum structural design such that bioavailability is maximized while off-target effects are minimized in conjunction to the construction of a reliable delivery system to protect the miRNAs from rapid degradation following delivery. For lncRNAs, the challenges are even more pronounced. The ncRNA fields have just begun to comprehend and investigate the crucial roles of lncRNAs in tumor biology, yet they still face challenges regarding tissue specificity, delivery systems, and targeting. CircRNAs are relatively new subjects of investigation, and their limited evolutionary conservation and challenges in production hinder clinical translation. However, their clinical potential may become clearer as additional circRNAs are characterized. Moving forward, these barriers need to be considered and solved through advancements in delivery systems, advances in molecular engineering, and synergistically coupling existing clinical strategies with ncRNA-based strategies. While the use of ncRNAs present difficulties for accomplishing clinical work, the potential of putting ncRNA-based strategies into practice in the clinic for cancer use is intriguing. Importantly, future research should prioritize the development of efficient and tumor-specific delivery systems, such as nanoparticle-based carriers and engineered exosomes, to enhance the stability, bioavailability, and targeted action of ncRNA therapeutics. These delivery platforms improve targeting efficiency by enhancing cellular uptake, protecting ncRNAs from nuclease degradation, and enabling tumor-specific delivery through ligand or antibody surface modifications. Moreover, bioinformatics platforms and computational tools have facilitated large-scale analysis of the tumor immune microenvironment, enabling the identification of immune-related genes and pathways involved in cancer progression and immunotherapy response [191]. Furthermore, utilizing combination therapies that incorporate angling the modulation of ncRNAs with the use of immune checkpoint inhibitors, such as blocking PD-1 or PD-L1, may help to address challenges with resistance to treatment and improve patient outcomes. In addition, using multi-omics technologies combined with discovery of biomarkers through artificial intelligence may enable further refinement of patient population stratification and resulting pressure on delivering precision immunotherapies. Together, these integrative strategies may bridge the current gap between mechanistic discoveries and clinical application of ncRNA-based therapies in breast cancer.

## Data Availability

No datasets were generated or analysed during the current study.

## References

[1] Giaquinto AN, Sung H, Newman LA, Freedman RA, Smith RA, Star J, et al. Breast cancer statistics 2024. Cancer J Clin. 2024;74(6):477–95. 10.3322/caac.21863 39352042

[2] Komuravelli M, Gaddala A, Nama S, Matli P, Anchuri K. Awareness and Attitude Regarding the Risk Factors, and Self Examination for Breast Cancer in the Students of an Engineering College of South India. Eur J Mol Clin Med (EJMCM). 2022;9(02):2022.

[3] Sung H, Ferlay J, Siegel RL, Laversanne M, Soerjomataram I, Jemal A, et al. Global Cancer Statistics 2020: GLOBOCAN Estimates of Incidence and Mortality Worldwide for 36 Cancers in 185 Countries. CA Cancer J Clin. 2021;71(3):209–49. 10.3322/caac.21660 33538338

[4] Sun YS, Zhao Z, Yang ZN, Xu F, Lu HJ, Zhu ZY, et al. Risk Factors and Preventions of Breast Cancer. Int J Biol Sci. 2017;13(11):1387–97. 10.7150/ijbs.21635 PMC5715522 29209143

[5] Hassan MS, Ansari J, Spooner D, Hussain SA. Chemotherapy for breast cancer (Review). Oncol Rep. 2010;24(5):1121–31. 10.3892/or_00000963 20878101

[6] Clarke M, Collins R, Darby S, Davies C, Elphinstone P, Evans V, et al. Effects of radiotherapy and of differences in the extent of surgery for early breast cancer on local recurrence and 15-year survival: an overview of the randomised trials. Lancet. 2005;366(9503):2087–106. 10.1016/S0140-6736(05)67887-7 16360786

[7] Gartner R, Jensen MB, Nielsen J, Ewertz M, Kroman N, Kehlet H. Prevalence of and factors associated with persistent pain following breast cancer surgery. JAMA. 2009;302(18):1985–92. 10.1001/jama.2009.1568 19903919

[8] Fournier A, Berrino F, Clavel-Chapelon F. Unequal risks for breast cancer associated with different hormone replacement therapies: results from the E3N cohort study. Breast Cancer Res Treat. 2008;107(1):103–11. 10.1007/s10549-007-9523-x PMC2211383 17333341

[9] Miles DW, Towlson KE, Graham R, Reddish M, Longenecker BM, Taylor-Papadimitriou J, et al. A randomised phase II study of sialyl-Tn and DETOX-B adjuvant with or without cyclophosphamide pretreatment for the active specific immunotherapy of breast cancer. Br J Cancer. 1996;74(8):1292–6. 10.1038/bjc.1996.532 PMC2075933 8883420

[10] Huszno J, Budryk M, Kolosza Z, Nowara E. The risk factors of toxicity during chemotherapy and radiotherapy in breast cancer patients according to the presence of BRCA gene mutation. Contemp Oncol (Pozn). 2015;19(1):72–6. 10.5114/wo.2015.50014 PMC4507882 26199574

[11] Pavlopoulou A, Oktay Y, Vougas K, Louka M, Vorgias CE, Georgakilas AG. Determinants of resistance to chemotherapy and ionizing radiation in breast cancer stem cells. Cancer Lett. 2016;380(2):485–93. 10.1016/j.canlet.2016.07.018 27450721

[12] Li G, Wan Y, Jiao A, Jiang K, Cui G, Tang J, et al. Breaking boundaries: chronic diseases and the frontiers of immune microenvironments. Med Res. 2025;1(1):62–102.

[13] Djebali S, Davis CA, Merkel A, Dobin A, Lassmann T, Mortazavi A, et al. Landscape of transcription in human cells. Nature. 2012;489(7414):101–8. 10.1038/nature11233 PMC3684276 22955620

[14] Yang L, Froberg JE, Lee JT. Long noncoding RNAs: fresh perspectives into the RNA world. Trends Biochem Sci. 2014;39(1):35–43. 10.1016/j.tibs.2013.10.002 PMC3904784 24290031

[15] Huang T, Alvarez A, Hu B, Cheng SY. Noncoding RNAs in cancer and cancer stem cells. Chin J Cancer. 2013;32(11):582–93. 10.5732/cjc.013.10170 PMC3845549 24206916

[16] Xu J, Wu KJ, Jia QJ, Ding XF. Roles of miRNA and lncRNA in triple-negative breast cancer. J Zhejiang Univ Sci B. 2020;21(9):673–89. 10.1631/jzus.B1900709 PMC7519626 32893525

[17] Le P, Romano G, Nana-Sinkam P, Acunzo M. Non-Coding RNAs in Cancer Diagnosis and Therapy: Focus on Lung Cancer. Cancers (Basel). 2021;13(6). 10.3390/cancers13061372 PMC8003033 33803619

[18] Kamali AN, Bautista JM, Eisenhut M, Hamedifar H. Immune checkpoints and cancer immunotherapies: insights into newly potential receptors and ligands. Ther Adv Vaccines Immunother. 2023;11:25151355231192043. 10.1177/25151355231192043 PMC10469281 37662491

[19] Taefehshokr N, Baradaran B, Baghbanzadeh A, Taefehshokr S. Promising approaches in cancer immunotherapy. Immunobiology. 2020;225(2):151875. 10.1016/j.imbio.2019.11.010 31812343

[20] Dong H, Zhu G, Tamada K, Chen L. B7-H1, a third member of the B7 family, co-stimulates T-cell proliferation and interleukin-10 secretion. Nat Med. 1999;5(12):1365–9. 10.1038/70932 10581077

[21] Sun C, Mezzadra R, Schumacher TN. Regulation and Function of the PD-L1 Checkpoint. Immunity. 2018;48(3):434–52. 10.1016/j.immuni.2018.03.014 PMC7116507 29562194

[22] Keir ME, Butte MJ, Freeman GJ, Sharpe AH. PD-1 and its ligands in tolerance and immunity. Annu Rev Immunol. 2008;26:677–704. 10.1146/annurev.immunol.26.021607.090331 PMC10637733 18173375

[23] Freeman GJ, Long AJ, Iwai Y, Bourque K, Chernova T, Nishimura H, et al. Engagement of the PD-1 immunoinhibitory receptor by a novel B7 family member leads to negative regulation of lymphocyte activation. J Exp Med. 2000;192(7):1027–34. 10.1084/jem.192.7.1027 PMC2193311 11015443

[24] Poggio M, Hu T, Pai CC, Chu B, Belair CD, Chang A, et al. Suppression of Exosomal PD-L1 Induces Systemic Anti-tumor Immunity and Memory. Cell. 2019;177(2):414–27. e13. 10.1016/j.cell.2019.02.016 PMC6499401 30951669

[25] Dong H, Strome SE, Salomao DR, Tamura H, Hirano F, Flies DB, et al. Tumor-associated B7-H1 promotes T-cell apoptosis: a potential mechanism of immune evasion. Nat Med. 2002;8(8):793–800. 10.1038/nm730 12091876

[26] Martins F, Sofiya L, Sykiotis GP, Lamine F, Maillard M, Fraga M, et al. Adverse effects of immune-checkpoint inhibitors: epidemiology, management and surveillance. Nat Rev Clin Oncol. 2019;16(9):563–80. 10.1038/s41571-019-0218-0 31092901

[27] Yin Q, Wu L, Han L, Zheng X, Tong R, Li L, et al. Immune-related adverse events of immune checkpoint inhibitors: a review. Front Immunol. 2023;14:1167975. 10.3389/fimmu.2023.1167975 PMC10247998 37304306

[28] Li Y, Al Hallak MN, Philip PA, Azmi AS, Mohammad RM. Non-Coding RNAs in Pancreatic Cancer Diagnostics and Therapy: Focus on lncRNAs, circRNAs, and piRNAs. Cancers (Basel). 2021;13(16). 10.3390/cancers13164161 PMC8392713 34439315

[29] Liu CG, Li J, Xu Y, Li W, Fang SX, Zhang Q, et al. Long non-coding RNAs and circular RNAs in tumor angiogenesis: From mechanisms to clinical significance. Mol Ther Oncolytics. 2021;22:336–54. 10.1016/j.omto.2021.07.001 PMC8426176 34553023

[30] Ponting CP, Oliver PL, Reik W. Evolution and functions of long noncoding RNAs. Cell. 2009;136(4):629–41. 10.1016/j.cell.2009.02.006 19239885

[31] Li X, Yang L, Chen LL. The Biogenesis, Functions, and Challenges of Circular RNAs. Mol Cell. 2018;71(3):428–42. 10.1016/j.molcel.2018.06.034 30057200

[32] Gibb EA, Brown CJ, Lam WL. The functional role of long non-coding RNA in human carcinomas. Mol Cancer. 2011;10(1):38. 10.1186/1476-4598-10-38 PMC3098824 21489289

[33] Huang X, Song C, Zhang J, Zhu L, Tang H. Circular RNAs in breast cancer diagnosis, treatment and prognosis. Oncol Res. 2023;32(2):241–9. 10.32604/or.2023.046582 PMC10765117 38186573

[34] Kristensen LS, Andersen MS, Stagsted LVW, Ebbesen KK, Hansen TB, Kjems J. The biogenesis, biology and characterization of circular RNAs. Nat Rev Genet. 2019;20(11):675–91. 10.1038/s41576-019-0158-7 31395983

[35] O’Brien J, Hayder H, Zayed Y, Peng C. Overview of MicroRNA Biogenesis, Mechanisms of Actions, and Circulation. Front Endocrinol (Lausanne). 2018;9:402. 10.3389/fendo.2018.00402 PMC6085463 30123182

[36] Rodriguez A, Griffiths-Jones S, Ashurst JL, Bradley A. Identification of mammalian microRNA host genes and transcription units. Genome Res. 2004;14(10A):1902–10. 10.1101/gr.2722704 PMC524413 15364901

[37] Ruby JG, Jan CH, Bartel DP. Intronic microRNA precursors that bypass Drosha processing. Nature. 2007;448(7149):83–6. 10.1038/nature05983 PMC2475599 17589500

[38] Tanzer A, Stadler PF. Molecular evolution of a microRNA cluster. J Mol Biol. 2004;339(2):327–35. 10.1016/j.jmb.2004.03.065 15136036

[39] Bartel DP. MicroRNAs: target recognition and regulatory functions. Cell. 2009;136(2):215–33. 10.1016/j.cell.2009.01.002 PMC3794896 19167326

[40] Lim LP, Lau NC, Garrett-Engele P, Grimson A, Schelter JM, Castle J, et al. Microarray analysis shows that some microRNAs downregulate large numbers of target mRNAs. Nature. 2005;433(7027):769–73. 10.1038/nature03315 15685193

[41] Krek A, Grun D, Poy MN, Wolf R, Rosenberg L, Epstein EJ, et al. Combinatorial microRNA target predictions. Nat Genet. 2005;37(5):495–500. 10.1038/ng1536 15806104

[42] Broughton JP, Lovci MT, Huang JL, Yeo GW, Pasquinelli AE. Pairing beyond the Seed Supports MicroRNA Targeting Specificity. Mol Cell. 2016;64(2):320–33. 10.1016/j.molcel.2016.09.004 PMC5074850 27720646

[43] Gebert LFR, MacRae IJ. Regulation of microRNA function in animals. Nat Rev Mol Cell Biol. 2019;20(1):21–37. 10.1038/s41580-018-0045-7 PMC6546304 30108335

[44] Schraivogel D, Meister G. Import routes and nuclear functions of Argonaute and other small RNA-silencing proteins. Trends Biochem Sci. 2014;39(9):420–31. 10.1016/j.tibs.2014.07.004 25131816

[45] Peng Y, Croce CM. The role of MicroRNAs in human cancer. Signal Transduct Target Ther. 2016;1:15004. 10.1038/sigtrans.2015.4 PMC5661652 29263891

[46] Mizielska A, Dziechciowska I, Szczepanski R, Cisek M, Dabrowska M, Slezak J et al. Doxorubicin and Cisplatin Modulate miR-21, miR-106, miR-126, miR-155 and miR-199 Levels in MCF7, MDA-MB-231 and SK-BR-3 Cells That Makes Them Potential Elements of the DNA-Damaging Drug Treatment Response Monitoring in Breast Cancer Cells-A Preliminary Study. Genes (Basel). 2023;14(3). 10.3390/genes14030702 PMC10048428 36980974

[47] Ahmadi SM, Amirkhanloo S, Yazdian-Robati R, Ebrahimi H, Pirhayati FH, Almalki WH, et al. Recent advances in novel miRNA mediated approaches for targeting breast cancer. J Drug Target. 2023;31(8):777–93. 10.1080/1061186X.2023.2240979 37480323

[48] Lukasiewicz S, Czeczelewski M, Forma A, Baj J, Sitarz R, Stanislawek A. Breast Cancer-Epidemiology, Risk Factors, Classification, Prognostic Markers, and Current Treatment Strategies-An Updated Review. Cancers (Basel). 2021;13(17). 10.3390/cancers13174287 PMC8428369 34503097

[49] Wang J, Wang W, Ma F, Qian H. A hidden translatome in tumors-the coding lncRNAs. Sci China Life Sci. 2023;66(12):2755–72. 10.1007/s11427-022-2289-6 37154857

[50] van Heesch S, Witte F, Schneider-Lunitz V, Schulz JF, Adami E, Faber AB, et al. Translational Landsc Hum Heart Cell. 2019;178(1):242–60. e29. 10.1016/j.cell.2019.05.010 31155234

[51] Zhang Y. LncRNA-encoded peptides in cancer. J Hematol Oncol. 2024;17(1):66. 10.1186/s13045-024-01591-0 PMC11320871 39135098

[52] Statello L, Guo CJ, Chen LL, Huarte M. Gene regulation by long non-coding RNAs and its biological functions. Nat Rev Mol Cell Biol. 2021;22(2):96–118. 10.1038/s41580-020-00315-9 PMC7754182 33353982

[53] Chen YG, Satpathy AT, Chang HY. Gene regulation in the immune system by long noncoding RNAs. Nat Immunol. 2017;18(9):962–72. 10.1038/ni.3771 PMC9830650 28829444

[54] Bhan A, Mandal SS. LncRNA HOTAIR: A master regulator of chromatin dynamics and cancer. Biochim Biophys Acta. 2015;1856(1):151–64. 10.1016/j.bbcan.2015.07.001 PMC4544839 26208723

[55] Prensner JR, Iyer MK, Balbin OA, Dhanasekaran SM, Cao Q, Brenner JC, et al. Transcriptome sequencing across a prostate cancer cohort identifies PCAT-1, an unannotated lincRNA implicated in disease progression. Nat Biotechnol. 2011;29(8):742–9. 10.1038/nbt.1914 PMC3152676 21804560

[56] Gupta RA, Shah N, Wang KC, Kim J, Horlings HM, Wong DJ, et al. Long non-coding RNA HOTAIR reprograms chromatin state to promote cancer metastasis. Nature. 2010;464(7291):1071–6. 10.1038/nature08975 PMC3049919 20393566

[57] Mourtada-Maarabouni M, Pickard MR, Hedge VL, Farzaneh F, Williams GT. GAS5, a non-protein-coding RNA, controls apoptosis and is downregulated in breast cancer. Oncogene. 2009;28(2):195–208. 10.1038/onc.2008.373 18836484

[58] Kino T, Hurt DE, Ichijo T, Nader N, Chrousos GP. Noncoding RNA gas5 is a growth arrest- and starvation-associated repressor of the glucocorticoid receptor. Sci Signal. 2010;3(107):ra8. 10.1126/scisignal.2000568 PMC2819218 20124551

[59] Perez-Navarro Y, Salinas-Vera YM, Lopez-Camarillo C, Figueroa-Angulo EE, Alvarez-Sanchez ME. The role of long non-coding RNA NORAD in digestive system tumors. Noncoding RNA Res. 2025;10:55–62. 10.1016/j.ncrna.2024.09.002 PMC11406672 39296642

[60] Ransohoff JD, Wei Y, Khavari PA. The functions and unique features of long intergenic non-coding RNA. Nat Rev Mol Cell Biol. 2018;19(3):143–57. 10.1038/nrm.2017.104 PMC5889127 29138516

[61] Ma L, Bajic VB, Zhang Z. On the classification of long non-coding RNAs. RNA Biol. 2013;10(6):925–33. 10.4161/rna.24604 PMC4111732 23696037

[62] Perez CAG, Adachi S, Nong QD, Adhitama N, Matsuura T, Natsume T, et al. Sense-overlapping lncRNA as a decoy of translational repressor protein for dimorphic gene expression. PLoS Genet. 2021;17(7):e1009683. 10.1371/journal.pgen.1009683 PMC8351930 34319983

[63] Zhang X, Li H, Burnett JC, Rossi JJ. The role of antisense long noncoding RNA in small RNA-triggered gene activation. RNA. 2014;20(12):1916–28. 10.1261/rna.043968.113 PMC4238356 25344398

[64] Balas MM, Johnson AM. Exploring the mechanisms behind long noncoding RNAs and cancer. Noncoding RNA Res. 2018;3(3):108–17. 10.1016/j.ncrna.2018.03.001 PMC6114262 30175284

[65] Qiu M, Chen Y, Zeng C. Biological functions of circRNA in regulating the hallmarks of gastrointestinal cancer (Review). Int J Oncol. 2024;64(5). 10.3892/ijo.2024.5637 PMC10997371 38488023

[66] Liang Y, Liu N, Yang L, Tang J, Wang Y, Mei M. A Brief Review of circRNA Biogenesis, Detection, and Function. Curr Genomics. 2021;22(7):485–95. 10.2174/1389202922666210331130722 PMC8905635 35386433

[67] Guo JU, Agarwal V, Guo H, Bartel DP. Expanded identification and characterization of mammalian circular RNAs. Genome Biol. 2014;15(7):409. 10.1186/s13059-014-0409-z PMC4165365 25070500

[68] Fang N, Ding GW, Ding H, Li J, Liu C, Lv L, et al. Research Progress of Circular RNA in Gastrointestinal Tumors. Front Oncol. 2021;11:665246. 10.3389/fonc.2021.665246 PMC8082141 33937077

[69] Fu L, Jiang Z, Li T, Hu Y, Guo J. Circular RNAs in hepatocellular carcinoma: Functions and implications. Cancer Med. 2018;7(7):3101–9. 10.1002/cam4.1574 PMC6051148 29856133

[70] Piwecka M, Glazar P, Hernandez-Miranda LR, Memczak S, Wolf SA, Rybak-Wolf A et al. Loss of a mammalian circular RNA locus causes miRNA deregulation and affects brain function. Science. 2017;357(6357). 10.1126/science.aam8526 28798046

[71] Hansen TB, Jensen TI, Clausen BH, Bramsen JB, Finsen B, Damgaard CK, et al. Natural RNA circles function as efficient microRNA sponges. Nature. 2013;495(7441):384–8. 10.1038/nature11993 23446346

[72] Yang F, Hu A, Li D, Wang J, Guo Y, Liu Y, et al. Circ-HuR suppresses HuR expression and gastric cancer progression by inhibiting CNBP transactivation. Mol Cancer. 2019;18(1):158. 10.1186/s12943-019-1094-z PMC6852727 31718709

[73] Conn SJ, Pillman KA, Toubia J, Conn VM, Salmanidis M, Phillips CA, et al. The RNA binding protein quaking regulates formation of circRNAs. Cell. 2015;160(6):1125–34. 10.1016/j.cell.2015.02.014 25768908

[74] Yang Y, Fan X, Mao M, Song X, Wu P, Zhang Y, et al. Extensive translation of circular RNAs driven by N(6)-methyladenosine. Cell Res. 2017;27(5):626–41. 10.1038/cr.2017.31 PMC5520850 28281539

[75] Yang Y, Gao X, Zhang M, Yan S, Sun C, Xiao F, et al. Novel Role of FBXW7 Circular RNA in Repressing Glioma Tumorigenesis. J Natl Cancer Inst. 2018;110(3):304–15. 10.1093/jnci/djx166 PMC6019044 28903484

[76] Li Z, Huang C, Bao C, Chen L, Lin M, Wang X, et al. Exon-intron circular RNAs regulate transcription in the nucleus. Nat Struct Mol Biol. 2015;22(3):256–64. 10.1038/nsmb.2959 25664725

[77] Gualandi F, Trabanelli C, Rimessi P, Calzolari E, Toffolatti L, Patarnello T, et al. Multiple exon skipping and RNA circularisation contribute to the severe phenotypic expression of exon 5 dystrophin deletion. J Med Genet. 2003;40(8):e100. 10.1136/jmg.40.8.e100 PMC1735543 12920092

[78] Ashwal-Fluss R, Meyer M, Pamudurti NR, Ivanov A, Bartok O, Hanan M, et al. circRNA biogenesis competes with pre-mRNA splicing. Mol Cell. 2014;56(1):55–66. 10.1016/j.molcel.2014.08.019 25242144

[79] Zeng Y, Zou Y, Gao G, Zheng S, Wu S, Xie X, et al. The biogenesis, function and clinical significance of circular RNAs in breast cancer. Cancer Biol Med. 2021;19(1):14–29. 10.20892/j.issn.2095-3941.2020.0485 PMC8763001 34110722

[80] Kong Y, Yang L, Wei W, Lyu N, Zou Y, Gao G, et al. CircPLK1 sponges miR-296-5p to facilitate triple-negative breast cancer progression. Epigenomics. 2019;11(10):1163–76. 10.2217/epi-2019-0093 31337246

[81] Shi P, Sun J, He B, Song H, Li Z, Kong W, et al. Profiles of differentially expressed circRNAs in esophageal and breast cancer. Cancer Manag Res. 2018;10:2207–21. 10.2147/CMAR.S167863 PMC6061203 30087579

[82] Yin WB, Yan MG, Fang X, Guo JJ, Xiong W, Zhang RP. Circulating circular RNA hsa_circ_0001785 acts as a diagnostic biomarker for breast cancer detection. Clin Chim Acta. 2018;487:363–8. 10.1016/j.cca.2017.10.011 29045858

[83] Tang YY, Zhao P, Zou TN, Duan JJ, Zhi R, Yang SY, et al. Circular RNA hsa_circ_0001982 Promotes Breast Cancer Cell Carcinogenesis Through Decreasing miR-143. DNA Cell Biol. 2017;36(11):901–8. 10.1089/dna.2017.3862 28933584

[84] Zhang N, Bevan MJ. CD8(+) T cells: foot soldiers of the immune system. Immunity. 2011;35(2):161–8. 10.1016/j.immuni.2011.07.010 PMC3303224 21867926

[85] Francisco LM, Sage PT, Sharpe AH. The PD-1 pathway in tolerance and autoimmunity. Immunol Rev. 2010;236:219–42. 10.1111/j.1600-065X.2010.00923.x PMC2919275 20636820

[86] Wang X, Teng F, Kong L, Yu J. PD-L1 expression in human cancers and its association with clinical outcomes. Onco Targets Ther. 2016;9:5023–39. 10.2147/OTT.S105862 PMC4990391 27574444

[87] Pardoll DM. The blockade of immune checkpoints in cancer immunotherapy. Nat Rev Cancer. 2012;12(4):252–64. 10.1038/nrc3239 PMC4856023 22437870

[88] Mardi A, Aghebati-Maleki L. Bispecific antibodies (bsAbs) directed against PD-1/PD-L1 and CTLA-4; a mini review. Hum Antibodies. 2025;33(1–2):3–9. 10.1177/10932607251325751 40112316

[89] Yokosuka T, Takamatsu M, Kobayashi-Imanishi W, Hashimoto-Tane A, Azuma M, Saito T. Programmed cell death 1 forms negative costimulatory microclusters that directly inhibit T cell receptor signaling by recruiting phosphatase SHP2. J Exp Med. 2012;209(6):1201–17. 10.1084/jem.20112741 PMC3371732 22641383

[90] Zhang X, Schwartz JC, Guo X, Bhatia S, Cao E, Lorenz M, et al. Structural and functional analysis of the costimulatory receptor programmed death-1. Immunity. 2004;20(3):337–47. 10.1016/s1074-7613(04)00051-2 15030777

[91] Ohaegbulam KC, Assal A, Lazar-Molnar E, Yao Y, Zang X. Human cancer immunotherapy with antibodies to the PD-1 and PD-L1 pathway. Trends Mol Med. 2015;21(1):24–33. 10.1016/j.molmed.2014.10.009 PMC4282825 25440090

[92] Thompson RH, Gillett MD, Cheville JC, Lohse CM, Dong H, Webster WS, et al. Costimulatory B7-H1 in renal cell carcinoma patients: Indicator of tumor aggressiveness and potential therapeutic target. Proc Natl Acad Sci U S A. 2004;101(49):17174–9. 10.1073/pnas.0406351101 PMC534606 15569934

[93] Gompelmann D, Sarova P, Mosleh B, Papaporfyriou A, Oberndorfer F, Idzko M, et al. PD-L1 assessment in lung cancer biopsies-pitfalls and limitations. Int J Biol Markers. 2024;39(1):3–8. 10.1177/03936155231214273 38111297

[94] Latchman Y, Wood CR, Chernova T, Chaudhary D, Borde M, Chernova I, et al. PD-L2 is a second ligand for PD-1 and inhibits T cell activation. Nat Immunol. 2001;2(3):261–8. 10.1038/85330 11224527

[95] Schildberg FA, Klein SR, Freeman GJ, Sharpe AH. Coinhibitory Pathways in the B7-CD28 Ligand-Receptor Family. Immunity. 2016;44(5):955–72. 10.1016/j.immuni.2016.05.002 PMC4905708 27192563

[96] Gordon SR, Maute RL, Dulken BW, Hutter G, George BM, McCracken MN, et al. PD-1 expression by tumour-associated macrophages inhibits phagocytosis and tumour immunity. Nature. 2017;545(7655):495–9. 10.1038/nature22396 PMC5931375 28514441

[97] Ribas A, Wolchok JD. Cancer immunotherapy using checkpoint blockade. Science. 2018;359(6382):1350–5. 10.1126/science.aar4060 PMC7391259 29567705

[98] Singh S, Hassan D, Aldawsari HM, Molugulu N, Shukla R, Kesharwani P. Immune checkpoint inhibitors: a promising anticancer therapy. Drug Discov Today. 2020;25(1):223–9. 10.1016/j.drudis.2019.11.003 31738877

[99] McDermott DF, Sosman JA, Sznol M, Massard C, Gordon MS, Hamid O, et al. Atezolizumab, an Anti-Programmed Death-Ligand 1 Antibody, in Metastatic Renal Cell Carcinoma: Long-Term Safety, Clinical Activity, and Immune Correlates From a Phase Ia Study. J Clin Oncol. 2016;34(8):833–42. 10.1200/JCO.2015.63.7421 26755520

[100] D’Angelo SP, Russell J, Lebbe C, Chmielowski B, Gambichler T, Grob JJ, et al. Efficacy and Safety of First-line Avelumab Treatment in Patients With Stage IV Metastatic Merkel Cell Carcinoma: A Preplanned Interim Analysis of a Clinical Trial. JAMA Oncol. 2018;4(9):e180077. 10.1001/jamaoncol.2018.0077 PMC5885245 29566106

[101] Siu LL, Even C, Mesia R, Remenar E, Daste A, Delord JP, et al. Safety and Efficacy of Durvalumab With or Without Tremelimumab in Patients With PD-L1-Low/Negative Recurrent or Metastatic HNSCC: The Phase 2 CONDOR Randomized Clinical Trial. JAMA Oncol. 2019;5(2):195–203. 10.1001/jamaoncol.2018.4628 PMC6439564 30383184

[102] Kruger S, Ilmer M, Kobold S, Cadilha BL, Endres S, Ormanns S, et al. Advances in cancer immunotherapy 2019 - latest trends. J Exp Clin Cancer Res. 2019;38(1):268. 10.1186/s13046-019-1266-0 PMC6585101 31217020

[103] Bagir EK, Acikalin A, Avci A, Gumurdulu D, Paydas S. PD-1 and PD-L1 expression in thymic epithelial tumours and non-neoplastic thymus. J Clin Pathol. 2018;71(7):637–41. 10.1136/jclinpath-2017-204788 29439008

[104] Hamanishi J, Mandai M, Iwasaki M, Okazaki T, Tanaka Y, Yamaguchi K, et al. Programmed cell death 1 ligand 1 and tumor-infiltrating CD8 + T lymphocytes are prognostic factors of human ovarian cancer. Proc Natl Acad Sci U S A. 2007;104(9):3360–5. 10.1073/pnas.0611533104 PMC1805580 17360651

[105] Sun JY, Zhang D, Wu S, Xu M, Zhou X, Lu XJ, et al. Resistance to PD-1/PD-L1 blockade cancer immunotherapy: mechanisms, predictive factors, and future perspectives. Biomark Res. 2020;8(1):35. 10.1186/s40364-020-00212-5 PMC7450549 32864132

[106] Reck M, Rodriguez-Abreu D, Robinson AG, Hui R, Csoszi T, Fulop A, et al. Pembrolizumab versus Chemotherapy for PD-L1-Positive Non-Small-Cell Lung Cancer. N Engl J Med. 2016;375(19):1823–33. 10.1056/NEJMoa1606774 27718847

[107] Song M, Chen X, Wang L, Zhang Y. Future of anti-PD-1/PD-L1 applications: Combinations with other therapeutic regimens. Chin J Cancer Res. 2018;30(2):157–72. 10.21147/j.issn.1000-9604.2018.02.01 PMC5953954 29861603

[108] Iorio MV, Ferracin M, Liu CG, Veronese A, Spizzo R, Sabbioni S, et al. MicroRNA gene expression deregulation in human breast cancer. Cancer Res. 2005;65(16):7065–70. 10.1158/0008-5472.CAN-05-1783 16103053

[109] Tavazoie SF, Alarcon C, Oskarsson T, Padua D, Wang Q, Bos PD, et al. Endogenous human microRNAs that suppress breast cancer metastasis. Nature. 2008;451(7175):147–52. 10.1038/nature06487 PMC2782491 18185580

[110] Valastyan S, Reinhardt F, Benaich N, Calogrias D, Szasz AM, Wang ZC, et al. A pleiotropically acting microRNA, miR-31, inhibits breast cancer metastasis. Cell. 2009;137(6):1032–46. 10.1016/j.cell.2009.03.047 PMC2766609 19524507

[111] Azarbarzin S, Hosseinpour-Feizi MA, Banan Khojasteh SM, Baradaran B, Safaralizadeh R. MicroRNA – 383-5p restrains the proliferation and migration of breast cancer cells and promotes apoptosis via inhibition of PD-L1. Life Sci. 2021;267:118939. 10.1016/j.lfs.2020.118939 33359245

[112] Mardi A, Ghovahi A, Abbasvandi F, Amani D. Experimental Validation of miR-4443, miR-572, and miR-150-5p in Serum and Tissue of Breast Cancer Patients as a Potential Diagnostic Biomarker: A Study Based on Bioinformatics Prediction. Biochem Genet. 2026;64(1):1117–44. 10.1007/s10528-025-11057-8 40064800

[113] Naseri B, Mardi A, Shekari N, Shajari N, Abdolzadeh S, Khorramdelazad H, et al. Ambivalent roles of miRNAs in cancer development via modulating tumor-associated innate immune cells. Bioimpacts. 2025;15:31430. 10.34172/bi.31430 PMC12705287 41409584

[114] Shadbad MA, Safaei S, Brunetti O, Derakhshani A, Lotfinejad P, Mokhtarzadeh A et al. A Systematic Review on the Therapeutic Potentiality of PD-L1-Inhibiting MicroRNAs for Triple-Negative Breast Cancer: Toward Single-Cell Sequencing-Guided Biomimetic Delivery. Genes (Basel). 2021;12(8). 10.3390/genes12081206 PMC8391239 34440380

[115] Wang Q, Lin W, Tang X, Li S, Guo L, Lin Y et al. The Roles of microRNAs in Regulating the Expression of PD-1/PD-L1 Immune Checkpoint. Int J Mol Sci. 2017;18(12). 10.3390/ijms18122540 PMC5751143 29186904

[116] Grenda A, Krawczyk P. New Dancing Couple: PD-L1 and MicroRNA. Scand J Immunol. 2017;86(3):130–4. 10.1111/sji.12577 28675453

[117] Cioffi M, Trabulo SM, Vallespinos M, Raj D, Kheir TB, Lin ML, et al. The miR-25-93-106b cluster regulates tumor metastasis and immune evasion via modulation of CXCL12 and PD-L1. Oncotarget. 2017;8(13):21609–25. 10.18632/oncotarget.15450 PMC5400610 28423491

[118] Rahmani F, Tadayyon Tabrizi A, Hashemian P, Alijannejad S, Rahdar HA, Ferns GA, et al. Role of regulatory miRNAs of the Wnt/ beta-catenin signaling pathway in tumorigenesis of breast cancer. Gene. 2020;754:144892. 10.1016/j.gene.2020.144892 32534060

[119] Lesurf R, Aure MR, Mork HH, Vitelli V, Oslo Breast Cancer Research C, Lundgren S, et al. Molecular Features of Subtype-Specific Progression from Ductal Carcinoma In Situ to Invasive Breast Cancer. Cell Rep. 2016;16(4):1166–79. 10.1016/j.celrep.2016.06.051 27396337

[120] Aure MR, Vitelli V, Jernstrom S, Kumar S, Krohn M, Due EU, et al. Integrative clustering reveals a novel split in the luminal A subtype of breast cancer with impact on outcome. Breast Cancer Res. 2017;19(1):44. 10.1186/s13058-017-0812-y PMC5372339 28356166

[121] Aure MR, Jernstrom S, Krohn M, Vollan HK, Due EU, Rodland E, et al. Integrated analysis reveals microRNA networks coordinately expressed with key proteins in breast cancer. Genome Med. 2015;7(1):21. 10.1186/s13073-015-0135-5 PMC4396592 25873999

[122] Yang M, Xiao R, Wang X, Xiong Y, Duan Z, Li D, et al. MiR-93-5p regulates tumorigenesis and tumor immunity by targeting PD-L1/CCND1 in breast cancer. Ann Transl Med. 2022;10(4):203. 10.21037/atm-22-97 PMC8908185 35280383

[123] Deng S, Wang M, Wang C, Zeng Y, Qin X, Tan Y, et al. p53 downregulates PD-L1 expression via miR-34a to inhibit the growth of triple-negative breast cancer cells: a potential clinical immunotherapeutic target. Mol Biol Rep. 2023;50(1):577–87. 10.1007/s11033-022-08047-z 36352176

[124] Johannessen C, Moi L, Kiselev Y, Pedersen MI, Dalen SM, Braaten T, et al. Expression and function of the miR-143/145 cluster in vitro and in vivo in human breast cancer. PLoS ONE. 2017;12(10):e0186658. 10.1371/journal.pone.0186658 PMC5657998 29073169

[125] Hajibabaei S, Sotoodehnejadnematalahi F, Nafissi N, Zeinali S, Azizi M. Aberrant promoter hypermethylation of miR-335 and miR-145 is involved in breast cancer PD-L1 overexpression. Sci Rep. 2023;13(1):1003. 10.1038/s41598-023-27415-8 PMC9849328 36653507

[126] Dastmalchi N, Hosseinpourfeizi MA, Khojasteh SMB, Baradaran B, Safaralizadeh R. Tumor suppressive activity of miR-424-5p in breast cancer cells through targeting PD-L1 and modulating PTEN/PI3K/AKT/mTOR signaling pathway. Life Sci. 2020;259:118239. 10.1016/j.lfs.2020.118239 32784058

[127] Valovka T, Schonfeld M, Raffeiner P, Breuker K, Dunzendorfer-Matt T, Hartl M, et al. Transcriptional control of DNA replication licensing by Myc. Sci Rep. 2013;3:3444. 10.1038/srep03444 PMC3853707 24309437

[128] Carmeliet P. VEGF as a key mediator of angiogenesis in cancer. Oncology. 2005;69(Suppl 3):4–10. 10.1159/000088478 16301830

[129] Rasoolnezhad M, Safaralizadeh R, Hosseinpourfeizi MA, Banan-Khojasteh SM, Baradaran B. MiRNA-138-5p: A strong tumor suppressor targeting PD-L-1 inhibits proliferation and motility of breast cancer cells and induces apoptosis. Eur J Pharmacol. 2021;896:173933. 10.1016/j.ejphar.2021.173933 33545160

[130] Guo Y, Xie Y, Luo Y. The Role of Long Non-Coding RNAs in the Tumor Immune Microenvironment. Front Immunol. 2022;13:851004. 10.3389/fimmu.2022.851004 PMC8863945 35222443

[131] Schmitt AM, Chang HY. Long noncoding RNAs in cancer pathways. Cancer Cell. 2016;29(4):452–63. 10.1016/j.ccell.2016.03.010 PMC4831138 27070700

[132] Yang Q, Fu Y, Wang J, Yang H, Zhang X. Roles of lncRNA in the diagnosis and prognosis of triple-negative breast cancer. J Zhejiang Univ Sci B. 2023;24(12):1123–40. 10.1631/jzus.B2300067 PMC10710915 38057269

[133] Li X, Li N. LncRNAs on guard. Int Immunopharmacol. 2018;65:60–3. 10.1016/j.intimp.2018.09.031 30286432

[134] Yu WD, Wang H, He QF, Xu Y, Wang XC. Long noncoding RNAs in cancer-immunity cycle. J Cell Physiol. 2018;233(9):6518–23. 10.1002/jcp.26568 PMC13482439 29574911

[135] Wang J, Liu X, Wu H, Ni P, Gu Z, Qiao Y, et al. CREB up-regulates long non-coding RNA, HULC expression through interaction with microRNA-372 in liver cancer. Nucleic Acids Res. 2010;38(16):5366–83. 10.1093/nar/gkq285 PMC2938198 20423907

[136] Cesana M, Cacchiarelli D, Legnini I, Santini T, Sthandier O, Chinappi M, et al. A long noncoding RNA controls muscle differentiation by functioning as a competing endogenous RNA. Cell. 2011;147(2):358–69. 10.1016/j.cell.2011.09.028 PMC3234495 22000014

[137] Samir A, Tawab RA, El Tayebi HM. Long non-coding RNAs XIST and MALAT1 hijack the PD-L1 regulatory signaling pathway in breast cancer subtypes. Oncol Lett. 2021;22(2):593. 10.3892/ol.2021.12854 PMC8200942 34149904

[138] Zheng L, Zhang Y, Fu Y, Gong H, Guo J, Wu K et al. Long non-coding RNA MALAT1 regulates BLCAP mRNA expression through binding to miR-339-5p and promotes poor prognosis in breast cancer. Biosci Rep. 2019;39(2). 10.1042/BSR20181284 PMC6379223 30683807

[139] Adewunmi O, Shen Y, Zhang XH, Rosen JM. Targeted Inhibition of lncRNA Malat1 Alters the Tumor Immune Microenvironment in Preclinical Syngeneic Mouse Models of Triple-Negative Breast Cancer. Cancer Immunol Res. 2023;11(11):1462–79. 10.1158/2326-6066.CIR-23-0045 PMC10618655 37603945

[140] Salama EA, Adbeltawab RE, El Tayebi HM. XIST and TSIX: Novel Cancer Immune Biomarkers in PD-L1-Overexpressing Breast Cancer Patients. Front Oncol. 2019;9:1459. 10.3389/fonc.2019.01459 PMC6966712 31998636

[141] Huang YS, Chang CC, Lee SS, Jou YS, Shih HM. Xist reduction in breast cancer upregulates AKT phosphorylation via HDAC3-mediated repression of PHLPP1 expression. Oncotarget. 2016;7(28):43256–66. 10.18632/oncotarget.9673 PMC5190021 27248326

[142] Zheng R, Lin S, Guan L, Yuan H, Liu K, Liu C, et al. Long non-coding RNA XIST inhibited breast cancer cell growth, migration, and invasion via miR-155/CDX1 axis. Biochem Biophys Res Commun. 2018;498(4):1002–8. 10.1016/j.bbrc.2018.03.104 29550489

[143] Gayen S, Maclary E, Buttigieg E, Hinten M, Kalantry S. A Primary Role for the Tsix lncRNA in Maintaining Random X-Chromosome Inactivation. Cell Rep. 2015;11(8):1251–65. 10.1016/j.celrep.2015.04.039 PMC4449283 25981039

[144] Zhang M, Wang N, Song P, Fu Y, Ren Y, Li Z, et al. LncRNA GATA3-AS1 facilitates tumour progression and immune escape in triple-negative breast cancer through destabilization of GATA3 but stabilization of PD-L1. Cell Prolif. 2020;53(9):e12855. 10.1111/cpr.12855 PMC7507373 32687248

[145] Kamel NA, Abdelzaher E, Elgebaly O, Ibrahim SA. Reduced expression of GATA3 predicts progression in non-muscle invasive urothelial carcinoma of the urinary bladder. J Histotechnol. 2020;43(1):21–8. 10.1080/01478885.2019.1667126 31551051

[146] Yin G, Liu Z, Wang Y, Sun L, Wang L, Yao B, et al. ZNF503 accelerates aggressiveness of hepatocellular carcinoma cells by down-regulation of GATA3 expression and regulated by microRNA-495. Am J Transl Res. 2019;11(6):3426–37. PMC6614650 31312355

[147] Wang Q, Li G, Ma X, Liu L, Liu J, Yin Y, et al. LncRNA TINCR impairs the efficacy of immunotherapy against breast cancer by recruiting DNMT1 and downregulating MiR-199a-5p via the STAT1-TINCR-USP20-PD-L1 axis. Cell Death Dis. 2023;14(2):76. 10.1038/s41419-023-05609-2 PMC9892521 36725842

[148] Dong H, Han J, Chen X, Sun H, Han M, Wang W. LncRNA ZNF649-AS1 promotes trastuzumab resistance and TAM-dependent PD-L1 expression in breast cancer by regulating EXOC7 alternative splicing. Arch Biochem Biophys. 2024;761:110128. 10.1016/j.abb.2024.110128 39159899

[149] Sherafat NS, Mardi A, Absalan F, Mehmandar-Oskuie A. Circular RNAs as central regulators of metabolic reprogramming and immune responses in the breast cancer microenvironment: A comprehensive review. Biomed Pharmacother. 2025;193:118682. 10.1016/j.biopha.2025.118682 41175594

[150] Fu B, Liu W, Zhu C, Li P, Wang L, Pan L, et al. Circular RNA circBCBM1 promotes breast cancer brain metastasis by modulating miR-125a/BRD4 axis. Int J Biol Sci. 2021;17(12):3104–17. 10.7150/ijbs.58916 PMC8375234 34421353

[151] Li J, Dong X, Kong X, Wang Y, Li Y, Tong Y, et al. Circular RNA hsa_circ_0067842 facilitates tumor metastasis and immune escape in breast cancer through HuR/CMTM6/PD-L1 axis. Biol Direct. 2023;18(1):48. 10.1186/s13062-023-00397-3 PMC10436663 37592296

[152] Li CW, Lim SO, Xia W, Lee HH, Chan LC, Kuo CW, et al. Glycosylation and stabilization of programmed death ligand-1 suppresses T-cell activity. Nat Commun. 2016;7:12632. 10.1038/ncomms12632 PMC5013604 27572267

[153] Dong LF, Chen FF, Fan YF, Zhang K, Chen HH. circ-0000512 inhibits PD-L1 ubiquitination through sponging miR-622/CMTM6 axis to promote triple-negative breast cancer and immune escape. J Immunother Cancer. 2023;11(6). 10.1136/jitc-2022-005461 PMC10314703 37349124

[154] Liang L, Gao M, Li W, Tang J, He Q, Zeng F, et al. CircGSK3beta mediates PD-L1 transcription through miR-338-3p/PRMT5/H3K4me3 to promote breast cancer cell immune evasion and tumor progression. Cell Death Discov. 2024;10(1):426. 10.1038/s41420-024-02197-8 PMC11452702 39366935

[155] Zhang Z, Huo W, Li J. circATAD2 mitigates CD8(+) T cells antitumor immune surveillance in breast cancer via IGF2BP3/m(6)A/PD-L1 manner. Cancer Immunol Immunother. 2024;73(7):130. 10.1007/s00262-024-03705-6 PMC11096152 38748254

[156] Huang L, Ma J, Cui M. Circular RNA hsa_circ_0001598 promotes programmed death-ligand-1-mediated immune escape and trastuzumab resistance via sponging miR-1184 in breast cancer cells. Immunol Res. 2021;69(6):558–67. 10.1007/s12026-021-09237-w 34559381

[157] Zheng Y, Ren S, Zhang Y, Liu S, Meng L, Liu F, et al. Circular RNA circWWC3 augments breast cancer progression through promoting M2 macrophage polarization and tumor immune escape via regulating the expression and secretion of IL-4. Cancer Cell Int. 2022;22(1):264. 10.1186/s12935-022-02686-9 PMC9396792 35996149

[158] Zhang H, Gong L, Yu L, Xian C, Ma Z, Wang X, et al. Emerging roles of non-coding RNA derived from extracellular vesicles in regulating PD-1/PD-L1 pathway: insights into cancer immunotherapy and clinical applications. Cancer Cell Int. 2025;25(1):188. 10.1186/s12935-025-03809-8 PMC12103061 40410719

[159] Hsu C-Y, Hjazi A, Suliman M, Singh G, Arora V, Shakhlo A et al. Disrupting immune silence: Noncoding RNAs targeting the programmed cell death protein 1/programmed cell death ligand 1 axis in tumor immunity. J Pharmacol Exp Ther. 2026;393(4). 10.1016/j.jpet.2026.104316 41921343

[160] Yang S, Wang X, Zhou X, Hou L, Wu J, Zhang W, et al. ncRNA-mediated ceRNA regulatory network: Transcriptomic insights into breast cancer progression and treatment strategies. Biomed Pharmacother. 2023;162:114698. 10.1016/j.biopha.2023.114698 37060661

[161] Shinde SS, Ahmed S, Malik JA, Hani U, Khanam A, Ashraf Bhat F, et al. Therapeutic delivery of tumor suppressor miRNAs for breast cancer treatment. Biology. 2023;12(3):467. 10.3390/biology12030467 PMC10045434 36979159

[162] Deng Y, Zhao F, Zhang Z, Sun F, Wang M. Long noncoding RNA SNHG7 promotes the tumor growth and epithelial-to-mesenchymal transition via regulation of miR-34a signals in osteosarcoma. Cancer Biother Radiopharm. 2018;33(9):365–72. 10.1089/cbr.2018.2503 29989838

[163] Rupaimoole R, Slack FJ. MicroRNA therapeutics: towards a new era for the management of cancer and other diseases. Nat Rev Drug Discovery. 2017;16(3):203–22. 10.1038/nrd.2016.246 28209991

[164] Zhang S, Cheng Z, Wang Y, Han T. The risks of miRNA therapeutics: in a drug target perspective. Drug design, development and therapy. 2021:721 – 33. 10.2147/DDDT.S288859 PMC7910153 33654378

[165] Bereczki Z, Benczik B, Balogh OM, Marton S, Puhl E, Pétervári M, et al. Mitigating off-target effects of small RNAs: conventional approaches, network theory and artificial intelligence. Br J Pharmacol. 2025;182(2):340–79. 10.1111/bph.17302 39293936

[166] Mu D, Han B, Huang H, Zheng Y, Zhang J, Shi Y. Unraveling the advances of non-coding RNAs on the tumor microenvironment: innovative strategies for cancer therapies. J Translational Med. 2025;23(1):614. 10.1186/s12967-025-06629-6 PMC12131439 40457447

[167] Usman WM, Pham TC, Kwok YY, Vu LT, Ma V, Peng B, et al. Efficient RNA drug delivery using red blood cell extracellular vesicles. Nat Commun. 2018;9(1):2359. 10.1038/s41467-018-04791-8 PMC6004015 29907766

[168] Simion V, Henriet E, Juric V, Aquino R, Loussouarn C, Laurent Y, et al. Intracellular trafficking and functional monitoring of miRNA delivery in glioblastoma using lipopolyplexes and the miRNA-ON RILES reporter system. J Controlled Release. 2020;327:429–43. 10.1016/j.jconrel.2020.08.028 32853728

[169] Yang J, Liu F, Wang Y, Qu L, Lin A. LncRNAs in tumor metabolic reprogramming and immune microenvironment remodeling. Cancer Lett. 2022;543:215798. 10.1016/j.canlet.2022.215798 35738332

[170] Hussain MS, Agrawal M, Shaikh NK, Saraswat N, Bahl G, Maqbool Bhat M, et al. Beyond the genome: deciphering the role of MALAT1 in breast cancer progression. Curr Genom. 2024;25(5):343–57. 10.2174/0113892029305656240503045154 PMC11420562 39323624

[171] Moghaddasnejad MR, Keshavarz A, Mardi A, Sherafat NS, Aghebati-Maleki L, Mohammadi MH. LncRNAs as behind-the-scenes molecules in cancer progression through regulating tumor-associated innate immune system cells. Mol Biol Rep. 2025;52(1):449. 10.1007/s11033-025-10513-3 40338353

[172] Wang Q, Li X, Ren S, Su C, Li C, Li W, et al. HOTAIR induces EGFR-TKIs resistance in non-small cell lung cancer through epithelial-mesenchymal transition. Lung Cancer. 2020;147:99–105. 10.1016/j.lungcan.2020.06.037 32683208

[173] Shabna A, Bindhya S, Sidhanth C, Garg M, Ganesan TS. Long non-coding RNAs: fundamental regulators and emerging targets of cancer stem cells. Biochim et Biophys Acta (BBA)-Reviews Cancer. 2023;1878(3):188899. 10.1016/j.bbcan.2023.188899 37105414

[174] Chen Y, Li Z, Chen X, Zhang S. Long non-coding RNAs: from disease code to drug role. Acta Pharm Sinica B. 2021;11(2):340–54. 10.1016/j.apsb.2020.10.001 PMC7893121 33643816

[175] Shaath H, Vishnubalaji R, Elango R, Kardousha A, Islam Z, Qureshi R, et al. editors. Long non-coding RNA and RNA-binding protein interactions in cancer: experimental and machine learning approaches. Seminars in cancer biology. Elsevier; 2022. 10.1016/j.semcancer.2022.05.013 35643221

[176] Elguindy MM, Mendell JT. NORAD-induced Pumilio phase separation is required for genome stability. Nature. 2021;595(7866):303–8. 10.1038/s41586-021-03633-w PMC8266761 34108682

[177] Bagherzadeh M, Safarkhani M, Daneshgar H, Radmanesh F, Taghavimandi F, Ghadiri AM, et al. Magnetic carbon–based nanocomposite decorated with palladium complex for co-delivery of DOX/pCRISPR. J Drug Deliv Sci Technol. 2022;78:103917. 10.1038/s41598-022-16058-w PMC9287414 35840687

[178] Yuan J, Wang Y, Huang Y, Li S, Zhang X, Wu Z, et al. Investigating Novel therapeutic approaches for idiopathic short stature: targeting siRNA and growth hormone delivery to the growth plate using exosome nanoparticles. Adv Sci. 2024;11(24):2309559. 10.1002/advs.202309559 PMC11200009 38639394

[179] Liang D-M, Li Y-J, Zhang J-X, Shen H-H, Wu C-X, Xie N, et al. m6A-methylated KCTD21-AS1 regulates macrophage phagocytosis through CD47 and cell autophagy through TIPR. Commun Biology. 2024;7(1):215. 10.1038/s42003-024-05854-x PMC10881998 38383737

[180] Kristensen LS, Andersen MS, Stagsted LV, Ebbesen KK, Hansen TB, Kjems J. The biogenesis, biology and characterization of circular RNAs. Nat Rev Genet. 2019;20(11):675–91. 10.1038/s41576-019-0158-7 31395983

[181] Dai D, Zhang J, Mo Y, Song C, Liu L, Chen ZS, et al. CircPLK1 upregulates ETS1 to confer anthracycline resistance in triple-negative breast cancer. J Transl Int Med. 2025;13(3):267–80. 10.1515/jtim-2025-0029 PMC12392080 40896288

[182] Lei M, Zheng G, Ning Q, Zheng J, Dong D. Translation and functional roles of circular RNAs in human cancer. Mol Cancer. 2020;19(1):30. 10.1186/s12943-020-1135-7 PMC7023758 32059672

[183] Huang D, Zhu X, Ye S, Zhang J, Liao J, Zhang N, et al. Tumour circular RNAs elicit anti-tumour immunity by encoding cryptic peptides. Nature. 2024;625(7995):593–602. 10.1038/s41586-023-06834-7 38093017

[184] Lin A, Ye P, Li Z, Jiang A, Liu Z, Cheng Q, et al. Natural killer cell immune checkpoints and their therapeutic targeting in cancer treatment. Research. 2025;8:0723. 10.34133/research.0723 PMC12131497 40463500

[185] Li H, Peng K, Yang K, Ma W, Qi S, Yu X, et al. Circular RNA cancer vaccines drive immunity in hard-to-treat malignancies. Theranostics. 2022;12(14):6422. 10.7150/thno.77350 PMC9475446 36168634

[186] Zhang W, Xu C, Yang Z, Zhou J, Peng W, Zhang X, et al. Circular RNAs in tumor immunity and immunotherapy. Mol Cancer. 2024;23(1):171. 10.1186/s12943-024-02082-z PMC11337656 39169354

[187] Perenkov AD, Sergeeva AD, Vedunova MV, Krysko DV. In vitro transcribed RNA-based platform vaccines: past, present, and future. Vaccines. 2023;11(10):1600. 10.3390/vaccines11101600 PMC10610676 37897003

[188] Li Y, Wang Z, Gao P, Cao D, Dong R, Zhu M, et al. CircRHBDD1 promotes immune escape via IGF2BP2/PD-L1 signaling and acts as a nanotherapeutic target in gastric cancer. J Translational Med. 2024;22(1):704. 10.1186/s12967-024-05498-9 PMC11289934 39080693

[189] Hu Z, Chen G, Zhao Y, Gao H, Li L, Yin Y, et al. Exosome-derived circCCAR1 promotes CD8 + T-cell dysfunction and anti-PD1 resistance in hepatocellular carcinoma. Mol Cancer. 2023;22(1):55. 10.1186/s12943-023-01759-1 PMC10024440 36932387

[190] Bao Q, Zhang H, Chen P, Wu S, Zou Y, Wang H, et al. The Dual Roles of Circular RNAs in Breast Cancer Distant Metastasis and Their Clinical Applications. J Cancer. 2025;16(10):3270–82. 10.7150/jca.111680 PMC12305621 40740236

[191] Liu L, Xie Y, Yang H, Lin A, Dong M, Wang H, et al. HPVTIMER: A shiny web application for tumor immune estimation in human papillomavirus-associated cancers. Imeta. 2023;2(3):e130. 10.1002/imt2.130 PMC10989930 38867938

[192] Robainas M, Otano R, Bueno S, Ait-Oudhia S. Understanding the role of PD-L1/PD1 pathway blockade and autophagy in cancer therapy. Onco Targets Ther. 2017;10:1803–7. 10.2147/OTT.S132508 PMC5370068 28367063

[193] Wu F, Yin Z, Yang L, Fan J, Xu J, Jin Y, et al. Smoking Induced Extracellular Vesicles Release and Their Distinct Properties in Non-Small Cell Lung Cancer. J Cancer. 2019;10(15):3435–43. 10.7150/jca.30425 PMC6603414 31293647

[194] Wang R, Huang Z, Qian C, Wang M, Zheng Y, Jiang R, et al. LncRNA WEE2-AS1 promotes proliferation and inhibits apoptosis in triple negative breast cancer cells via regulating miR-32-5p/TOB1 axis. Biochem Biophys Res Commun. 2020;526(4):1005–12. 10.1016/j.bbrc.2020.01.170 32307083

[195] Beltran-Anaya FO, Romero-Cordoba S, Rebollar-Vega R, Arrieta O, Bautista-Pina V, Dominguez-Reyes C, et al. Expression of long non-coding RNA ENSG00000226738 (LncKLHDC7B) is enriched in the immunomodulatory triple-negative breast cancer subtype and its alteration promotes cell migration, invasion, and resistance to cell death. Mol Oncol. 2019;13(4):909–27. 10.1002/1878-0261.12446 PMC6441920 30648789

[196] Wang S, Ke H, Zhang H, Ma Y, Ao L, Zou L, et al. LncRNA MIR100HG promotes cell proliferation in triple-negative breast cancer through triplex formation with p27 loci. Cell Death Dis. 2018;9(8):805. 10.1038/s41419-018-0869-2 PMC6057987 30042378

